# Global burden, trends and health inequalities of stroke attributable to household air pollution, 1990–2021: a decomposition and prediction analysis

**DOI:** 10.3389/fpubh.2025.1625842

**Published:** 2025-09-11

**Authors:** Xiaoqing Xia, Jing Yu, Deji Suona, Hong Zhi, Yongchen Hao, Lina Wang

**Affiliations:** ^1^Key Laboratory of Environmental Medicine Engineering of Ministry of Education, Department of Epidemiology & Biostatistics, School of Public Health, Southeast University, Nanjing, China; ^2^Department of Cardiology, ZhongDa Hospital, Southeast University, Nanjing, China; ^3^Center for Clinical and Epidemiological Research, Beijing An Zhen Hospital, Capital Medical University, Beijing Institute of Heart, Lung and Blood Vessel Diseases, Beijing, China

**Keywords:** stroke, household air pollution, trends, health inequalities, burden of disease

## Abstract

**Objective:**

Exposure to household air pollution from solid fuels (HAP) increases stroke risk, affecting approximately 3 billion people worldwide and posing a significant challenge to public health. This study assessed trends in the HAP-related global stroke burden from 1990 to 2021 and quantified associated health inequalities.

**Methods:**

Data on HAP-attributable stroke disability-adjusted life years (DALYs) and deaths from 1990 to 2021 at global, regional, and national levels were derived from the Global Burden of Disease (GBD) 2021. The estimated annual percentage change (EAPC) was employed to evaluate temporal trends. Decomposition analysis elucidated the primary drivers of burden changes. Cross-country inequality was examined by incorporating the Sociodemographic Index (SDI), and future disease burden was projected.

**Results:**

From 1990 to 2021, the age-standardized rates (ASR) of DALYs and deaths from HAP-related stroke declined globally, although significant geographic heterogeneity persisted. Compared with high SDI regions, lower SDI areas bore a heavier burden, with countries such as Zimbabwe and Lesotho experiencing the most notable increases. Furthermore, the share of global ischemic stroke burden increased, whereas intracerebral hemorrhage remained the dominant contributor. Decomposition analysis revealed that population growth consistently served as the main driver of burden increases in low-middle and low SDI areas. Notably, stroke burden was disproportionately concentrated in lower SDI nations, especially for the subarachnoid hemorrhage subtype. Projections suggested that by 2035, the global HAP-related stroke burden would increase, with the ischemic stroke subtype exhibiting the greatest growth.

**Conclusion:**

Although the global HAP-related stroke burden has reduced, significant regional and population disparities persist, along with severe health inequities. Therefore, emphasis should be placed on improving household energy supply and healthcare resource allocation in low-development regions to reduce preventable health inequities.

## 1 Introduction

Stroke is an acute cerebrovascular disorder caused by the rupture or occlusion of cerebral blood vessels, which leads to brain tissue damage. It is characterized by high recurrence, mortality, and disability rates, affecting nearly one-quarter of the global population during their lifetime (1). According to the 2021 Global Burden of Disease (GBD) dataset, stroke ranked among the leading global causes of mortality and disability (2). It is estimated that, excluding inflation, the global economic cost of stroke could range from $880 billion to $2.21 trillion by 2050 (3). Epidemiological evidence indicates that several risk factors, including air pollution, alcohol consumption, smoking, and dietary habits, play a crucial role in stroke pathogenesis (4–6). Controlling modifiable risk factors can effectively reduce stroke incidence throughout an individual’s lifespan, thereby alleviating the global health and economic burdens of stroke (3).

Globally, nearly 3 billion people remain exposed to household air pollution from solid fuels (HAP), which continues to be a significant contributor to stroke-related mortality, particularly in regions dependent on solid fuels (7, 8). HAP primarily refers to exposure to particulate matter with an aerodynamic diameter ≤2.5 μm (PM_2.5_) generated through the combustion of solid fuels, including coal and charcoal (4). In 2021, HAP caused approximately 3.1 million deaths and 111.5 million disability-adjusted life years (DALYs) globally, posing a significant global health threat (5). HAP severely affects the health of residents in underdeveloped regions and rural areas, serving as the primary risk factor for disease burden in low socio-demographic index (SDI) regions (4, 9). In countries where solid fuels are extensively utilized for cooking, heating, and lighting, poorly ventilated environments with incomplete combustion produce significant HAP (10, 11). A cohort study from several South Asian countries indicates that HAP, a modifiable risk factor, contributes 6.1% to the population-attributable fraction (PAF) for cardiovascular diseases (12).

Therefore, a systematic analysis of the HAP-related stroke burden at global, regional, and national levels, along with its spatiotemporal heterogeneity, is essential. However, comprehensive evaluations of the global stroke burden attributable to HAP remain limited, especially concerning recent spatiotemporal trends. This study utilized high-quality data from the GBD 2021 to summarize the HAP-related stroke burden by age, sex, region, country, and SDI, and to assess spatiotemporal trends over the past 32 years. We employed decomposition analysis to explore the dynamic changes in stroke burden across SDI-diverse regions and identify the key driving factors. Cross-country inequality analysis provided a detailed quantitative assessment of the global HAP-related stroke burden, while the Bayesian Age-Period-Cohort (BAPC) model projected future changes. The findings provide comprehensive scientific evidence to shape public health strategies and enhance medical resource distribution, offering new insights into advancing the Sustainable Development Goals (SDGs).

## 2 Materials and methods

### 2.1 Data source

The GBD 2021 is the largest and latest epidemiological assessment, covering 371 diseases and injuries at global, regional, and national levels (2). It draws on 100,983 distinct data sources, including censuses, health service utilization records, and other demographic and health system data (4). Advanced statistical models, including Bayesian meta-regression (e.g., DisMod-MR 2.1), were applied to guarantee internal coherence and produce highly accurate, cross-dimensionally comparable disease burden estimates across regions, time periods, age groups, and sexes (13).

For this study, all data were sourced from the GBD Results Tool.^1^ Consistent with our research objectives, we collected deaths and DALYs data related to HAP-induced stroke for both sexes aged 25 and older. The data were categorized into 13 age brackets, 5 SDI regions, 21 GBD regions, and 204 countries and territories.

### 2.2 Definitions

SDI serves as an indicator of the comprehensive development level of countries or regions. It is calculated using the total fertility rate for individuals under 25, the average educational attainment for those aged 15 and older, and per capita income, producing a value between 0 and 1. This study classified countries into five SDI categories—high, high-middle, middle, low-middle, and low to compare the HAP-attributable stroke burden across different socioeconomic settings globally.

In GBD 2021, HAP refers to PM_2.5_ produced by burning solid cooking fuels, including coal, charcoal, wood, dung, and crop residues (4). HAP exposure is influenced by two factors: the proportion of the population that utilizes solid fuels and the corresponding PM_2.5_ exposure level within this group. The main sources of exposure data include household surveys, the World Health Organization (WHO)’s Household Energy Database, and other relevant datasets (14). Compared with GBD 2010, the latest GBD study considers fuel type and extracts micro-level data when possible to improve the consistency of survey responses regarding fuel categories (14).

According to the WHO’s clinical criteria, stroke is defined as the rapid onset of focal cerebral dysfunction, with symptoms persisting for more than 24 h or leading to death (15). GBD 2021 classifies stroke into three pathological subtypes: ischemic stroke (IS), defined as neurological dysfunction caused by localized infarction in the brain, spinal cord, or retina; intracerebral hemorrhage (ICH), defined as focal intracranial bleeding resulting from non-traumatic vascular rupture; and subarachnoid hemorrhage (SAH) refers to a non-traumatic stroke caused by bleeding into the subarachnoid space.

### 2.3 Statistical analysis

To control for demographic heterogeneity across and within populations over time, this study utilized age-standardized mortality rates (ASMR) and DALY rates (ASDR) to evaluate the global stroke burden attributable to HAP. Additionally, we applied the estimated annual percentage change (EAPC) to assess temporal trends in age-standardized rates (ASRs). A regression line was fitted to the natural logarithm of the ASR: ln (*ASR*) = *α* + *βx* + *Ɛ*, where x denotes the calendar year. The EAPC and its 95% confidence intervals (CIs) were calculated from the regression coefficient using the formula: *EAPC* = 100 × (exp(*β*) − 1). If the EAPC and lower CI boundary was > 0, the ASR was considered to have increased over the specified period. In contrast, if the EAPC and upper CI boundary was < 0, the ASR was considered to have decreased. In all other cases, the ASR was considered stable.

### 2.4 Decomposition analysis

The Das-Gupta decomposition method was adopted to assess 32-year changes in the HAP-attributable stroke burden, which was partitioned into three components: epidemiological changes, population growth, and aging (16). Here, epidemiological changes refer to variations in age-specific stroke DALY rates related to HAP over time, after removing the effects of changes in total population size and age structure. These variations may reflect changes in disease incidence, case fatality, healthcare access, and other population health determinants (17). This approach enabled the assessment of each variable’s independent contribution to changes in disease burden, helping identify potential driving factors behind the global HAP-attributable stroke burden.

### 2.5 Cross-country inequality analysis

We employed two WHO-defined indices, the slope index of inequality (SII) and the concentration index (CI), to quantify both absolute and relative inequalities in the stroke burden attributable to HAP across countries (18). We calculated the SII using regression analysis, with each country’s DALY rate as the dependent variable and the midpoint of the cumulative population distribution ranked by SDI as the independent variable. CI was computed by numerically integrating area under Lorenz curve, representing alignment between cumulative DALY proportion and cumulative population ranked by SDI (19).

### 2.6 Predictive analysis

The above analysis primarily examined the burden of HAP-attributable stroke between 1990 and 2021. To support precise public health strategies and efficient healthcare resource allocation, we applied the BAPC model to project future burden through 2035. The model employed the integrated nested Laplace approximation (INLA) method to approximate the marginal posterior distribution, which effectively alleviated the mixing and convergence issues typically encountered in traditional Bayesian Markov chain Monte Carlo sampling methods (20). Using GBD 2021 estimates, demographic forecasts, and incorporating age, period, and cohort influences, the BAPC model more accurately predicted future disease burden trends. Meanwhile, to assess the robustness of projections through 2035, sensitivity analyses were conducted using the upper and lower bounds of the 95% uncertainty intervals (95% UI) provided by the GBD data.

### 2.7 Statistical significance and software

All analyses and visualizations were performed with R (version 4.4.1) and Stata (version 18.0). A two-sided *p* < 0.05 was considered statistically significant.

## 3 Results

### 3.1 Global burden of stroke attributed to HAP in 2021

**Figure 1:**
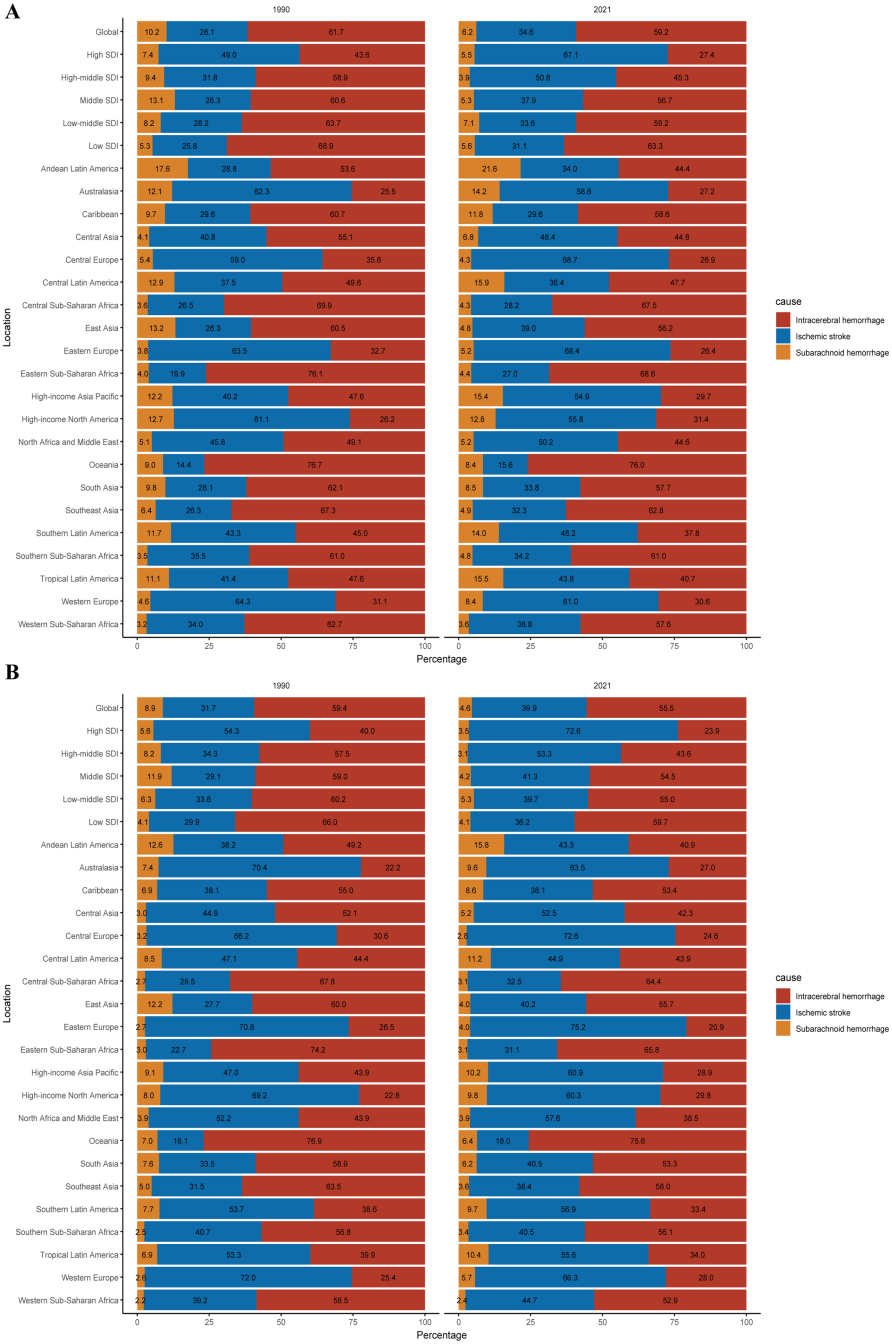
Contributions of ICH, IS, and SAH to the absolute numbers of stroke DALYs **(A)** and deaths **(B)** attributable to HAP globally and by region, in 1990 and 2021; ICH, intracerebral hemorrhage; IS, ischemic stroke; SAH, subarachnoid hemorrhage; DALYs, disability-adjusted life years.

In 2021, stroke caused approximately 160.5 million DALYs and 7.3 million deaths worldwide (5). That same year, stroke attributable to HAP led to 18.2 million DALYs and 0.76 million deaths, representing 11.34 and 10.46% of the total stroke burden, respectively (Supplementary Table S1). ICH accounted for more than half of stroke-induced deaths (55.5%) and DALYs (59.2%; Figure 1). Among the three stroke subtypes, ICH posed the highest burden (ASDR = 123.61 per 100,000, 95% UI: 72.34, 205.7; ASMR = 4.90 per 100,000, 95% UI: 2.75, 8.38), followed by IS and SAH.

In 2021, the HAP-induced stroke burden varied markedly across SDI regions. High and high-middle SDI regions experienced significantly lower burden compared to the others, with the greatest burden observed in low SDI regions (ASDR: 953.74 per 100,000; 95% UI: 747.82, 1144.44; ASMR: 44.12 per 100,000; 95% UI: 34.66, 53.28). ICH constituted the largest proportion of stroke-related deaths and DALYs in low SDI regions, whereas IS dominated the stroke burden in high and high-middle SDI areas.

Geographically, Oceania demonstrated the highest HAP-induced stroke burden (ASDR = 1297.23 per 100,000, 95% UI: 905.22, 1724.07; ASMR = 57.75 per 100,000, 95% UI: 40.13, 76.4), followed by Eastern and Central Sub-Saharan Africa. In contrast, Australasia exhibited the lowest burden (ASDR = 0.02 per 100,000, 95% UI: 0, 0.12; ASMR = 0 per 100,000, 95% UI: 0, 0.01), followed by High-income North America and the High-income Asia Pacific. ICH was the major contributor to HAP-related stroke DALYs in 11 GBD regions, representing 68.6% of stroke DALYs in Eastern Sub-Saharan Africa. IS predominated as the contributor to HAP-related stroke deaths in 12 GBD regions, representing 75.2% of stroke deaths in Western Europe.

**Figure 2:**
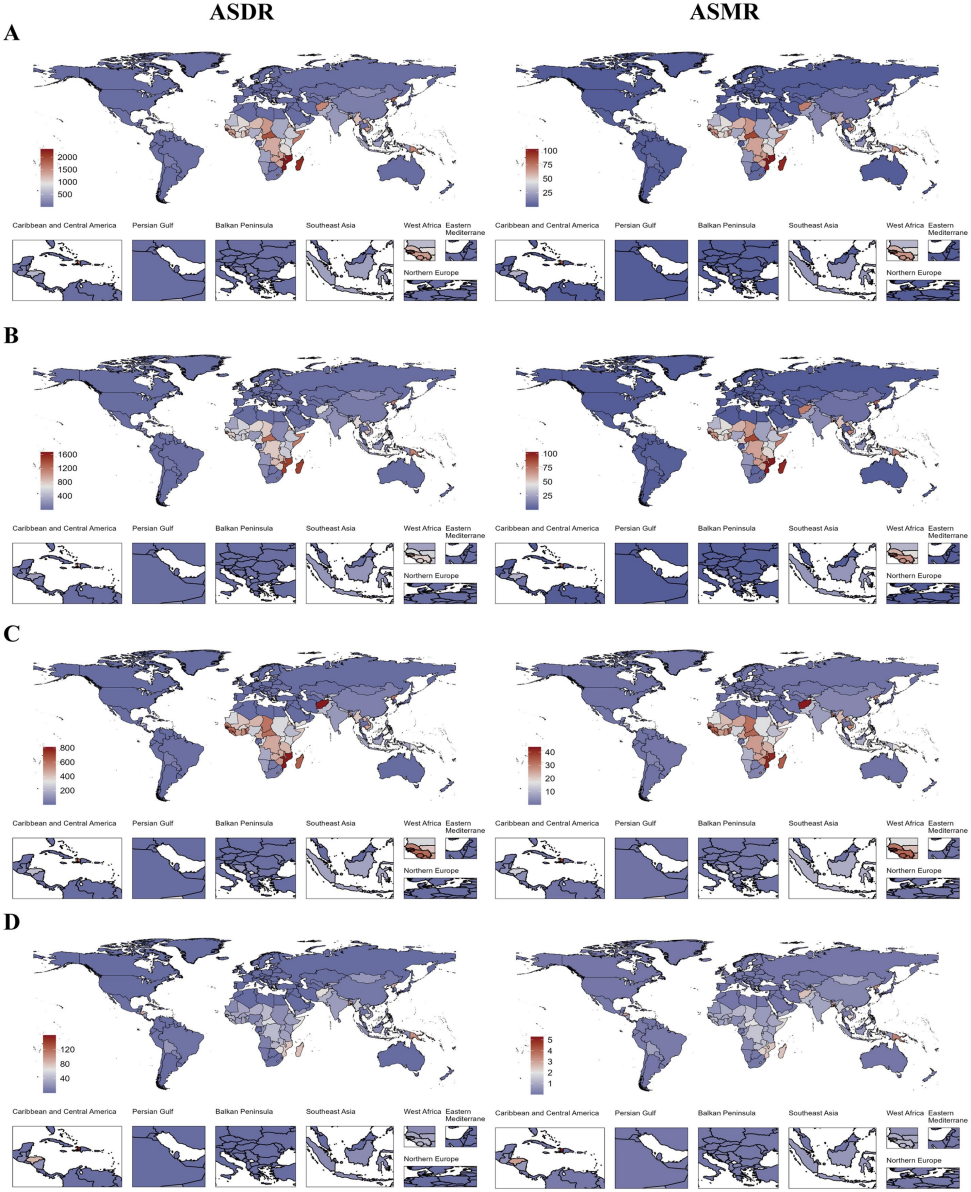
ASDR and ASMR of total stroke **(A)**, ICH **(B)**, IS **(C)**, and SAH **(D)** attributable to HAP at the national level in 2021; ASDR, age-standardized DALYs rate; ASMR, age-standardized mortality rate; HAP, household air pollution from solid fuels; ICH, intracerebral hemorrhage; IS, ischemic stroke; and SAH, subarachnoid hemorrhage.

In 2021, substantial heterogeneity was observed in the ASDR and ASMR for stroke across different countries. Mozambique exhibited the highest burden, with ASDR of 2328.45 per 100,000 (95% UI: 1661.84, 2986.85) and ASMR of 102.96 per 100,000 (95% UI: 74.43, 133.70), followed by the Solomon Islands and Madagascar (Figure 2). Regarding the three stroke subtypes, the Solomon Islands had the highest burden of ICH, Guinea-Bissau had the highest burden of IS, and Haiti had the highest burden of SAH.

### 3.2 Global stroke burden attributed to HAP by sex and age

In 2021, the ASDR and ASMR of stroke attributable to HAP were higher in males than females (Supplementary Table S1). As shown in Supplementary Figure S1, the death and DALY rates for HAP-related stroke increased with age. Moreover, age-specific death and DALY rates for HAP-related stroke remained consistently higher in males than in females.

### 3.3 Temporal trends of stroke burden attributed to HAP from 1990 to 2021

Globally, the EAPC of ASDR and ASMR for HAP-related stroke decreased by −4.38 (95% CI: −4.75, −4.00) and −4.46 (95% CI: −4.88, −4.04), respectively, accompanied by a reduction in absolute numbers (Supplementary Table S1). The burden of all three stroke subtypes exhibited a declining trend, with IS experiencing the slowest reduction. The proportion of the stroke burden due to ICH and SAH declined to varying extents, while that of IS increased (Figure 1).

**Figure 3:**
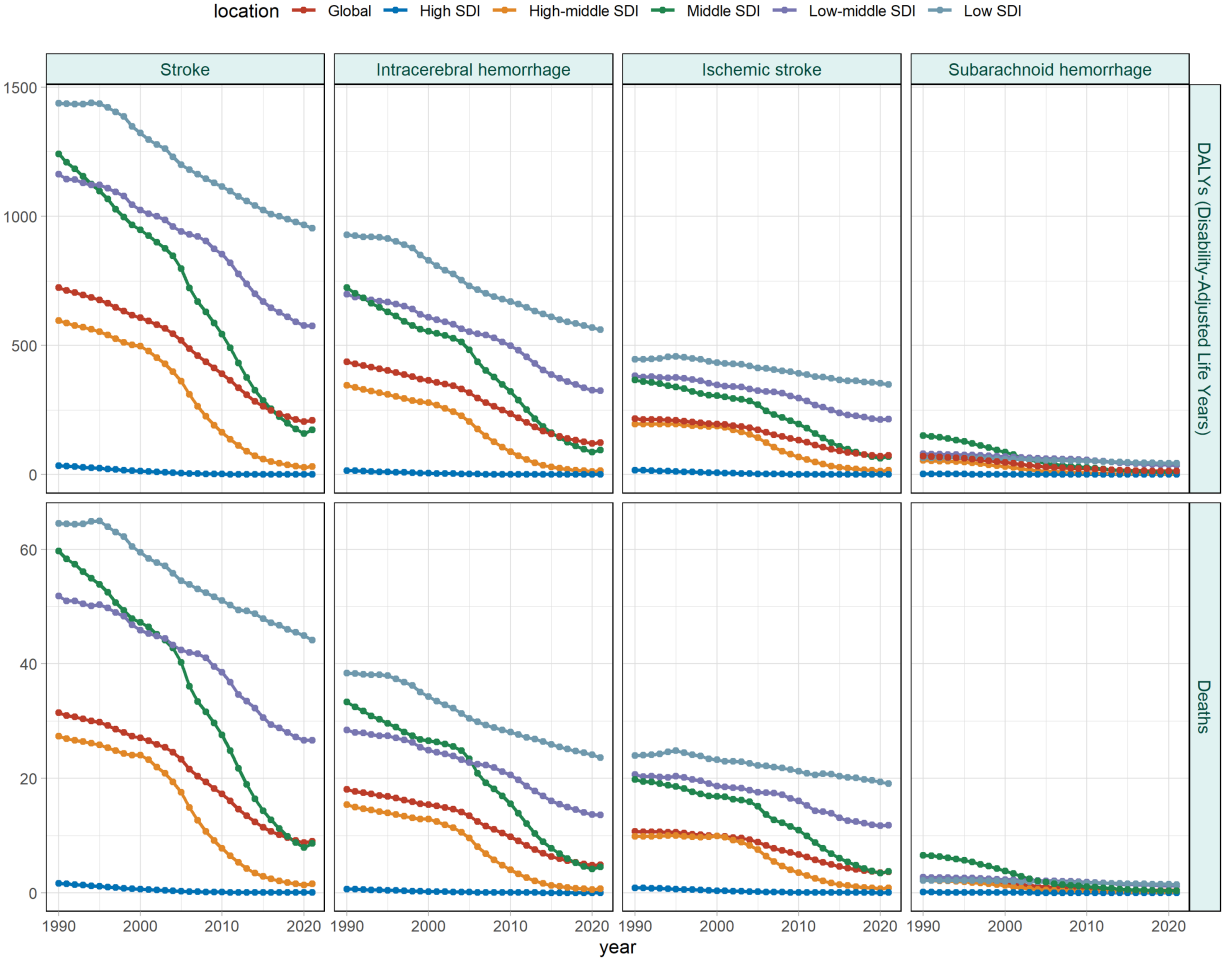
Temporal trends in ASDR and ASMR of stroke and its subtypes attributable to HAP by SDI region from 1990 to 2021; ASDR, age-standardized DALY rate; ASMR, age-standardized mortality rate; SDI, sociodemographic index; HAP, household air pollution from solid fuels.

The stroke burden declined to different degrees across all SDI regions, with the slowest reduction noted in low SDI regions [EAPC in ASDR: −1.51, 95% CI: −1.58, −1.44; EAPC in ASMR: −1.36, 95% CI: −1.42, −1.30] (Supplementary Table S1; Figure 3). Similarly, low SDI regions experienced the slowest decrease across all three stroke subtypes. IS contributed an increasing share to the total stroke burden across all SDI regions, with the largest increase occurring in high and high-middle SDI regions (Figure 1).

At different geographical levels, the stroke burden declined most rapidly in the High-income Asia Pacific (EAPC in ASDR: -14.96, 95% CI: −16.2, −13.7; EAPC in ASMR: −15.09, 95% CI: −16.29, −13.88), whereas the slowest reduction occurred in Oceania (EAPC in ASDR: −1.20, 95% CI: −1.23, −1.16; EAPC in ASMR: −1.16, 95% CI: −1.21, −1.11; Supplementary Table S1). Similarly, the burden associated with ICH subtype decreased at the slowest rate in Oceania. In contrast, IS and SAH exhibited the slowest reductions in Eastern and Southern Sub-Saharan Africa, respectively (Supplementary Table S2).

More than 90% of countries underwent a decline in stroke burden over the past 32 years. However, Zimbabwe showed the most significant rise in ASDR (EAPC = 2.16, 95% CI: 1.54, 2.79), followed by the Northern Mariana Islands and Lesotho. The Northern Mariana Islands demonstrated the strongest increase in ASMR (EAPC = 2.11, 95% CI: 0.80, 3.44), followed by Zimbabwe and Lesotho (Supplementary Figure S2). Furthermore, these three countries continued to lead in terms of burden increase across different stroke subtypes.

### 3.4 Decomposition analysis

**Figure 4:**
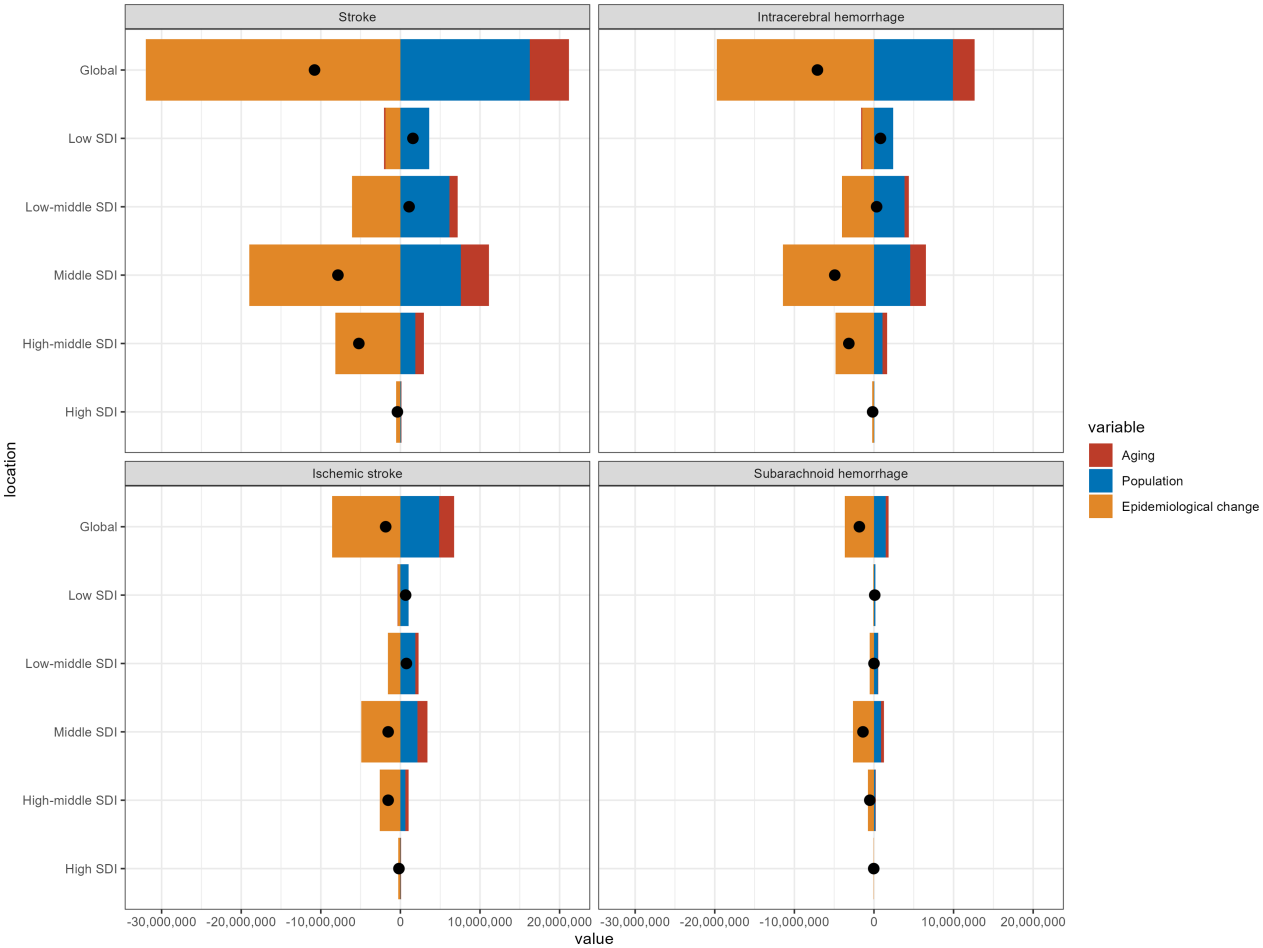
Changes in DALYs of stroke and its subtypes attributed to HAP according to aging, population growth and epidemiological change from 1990 to 2021 at the global level by SDI quintile; HAP, household air pollution from solid fuels; DALYs, disability-adjusted life years; SDI, sociodemographic index.

The findings illustrated the relative contributions of aging, population growth, and epidemiological changes to the HAP-induced stroke burden (Supplementary Table S4; Figure 4). Excluding the low and low-middle SDI regions, stroke DALYs declined globally and across other regions. Remarkably, the middle SDI regions showed the greatest reduction. Over the past 32 years, epidemiological changes accounted for 296.39% of the global decline in stroke burden, whereas population growth (−45.79%) and aging (−150.6%) had negative effects (Supplementary Table S3). The increase in DALYs in low SDI regions was primarily driven by population dynamics (231.43%), followed by epidemiological changes (−119.76%) and aging (−11.67%). Across all three stroke subtypes, burden increased in both low and low-middle SDI regions, with population growth solely driving the increase in low SDI regions.

### 3.5 Cross-country inequality analysis

**Figure 5:**
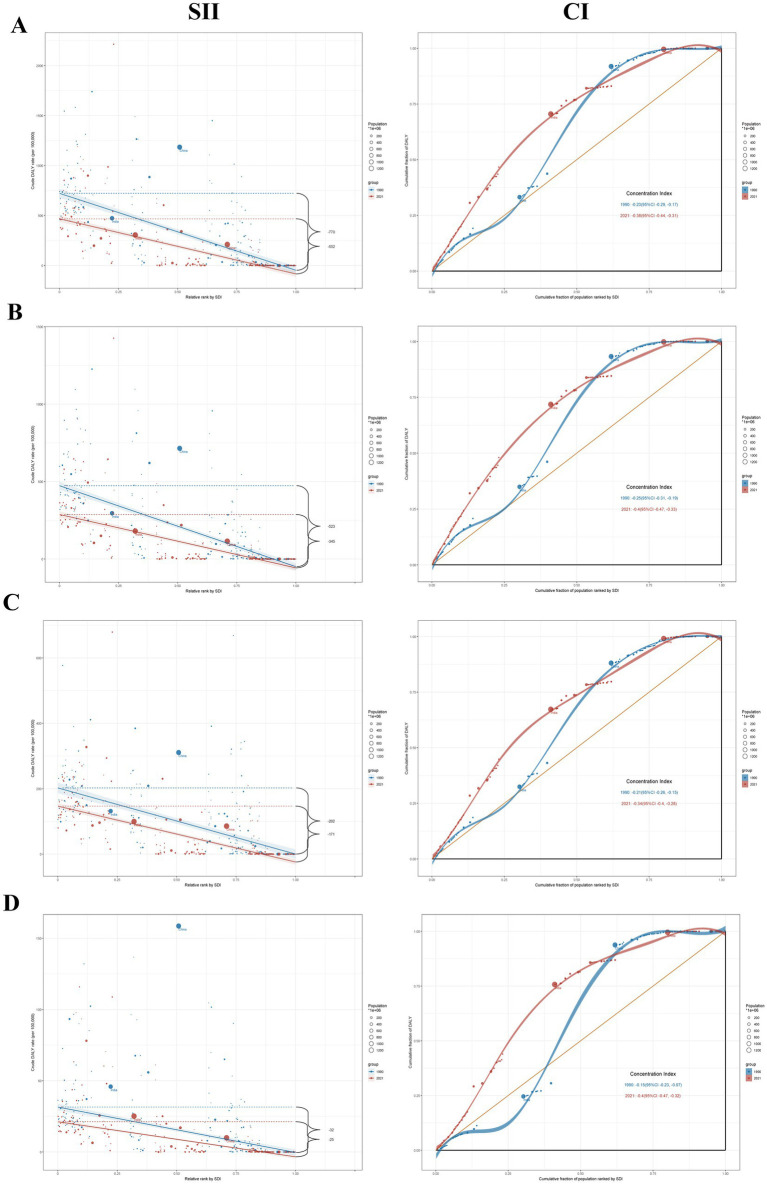
The SII and CI for DALYs of Stroke **(A)**, ICH **(B)**, IS **(C)**, and SAH **(D)** attributable to HAP worldwide in 1990 and 2021; DALYs, disability-adjusted life years; SII; slope index of inequality; CI; concentration indexes; ICH, intracerebral hemorrhage; IS, ischemic stroke; SAH, subarachnoid hemorrhage.

The SII for DALYs (per 100,000 population) was −769.9 (95% CI: −856.0, −683.0) in 1990 and −551.6 (95% CI: −613.1, −490.1) in 2021 (Supplementary Table S5; Figure 5). This decline indicated a reduction in the absolute disparity in HAP-induced stroke burden between low and high SDI countries over the period. However, the CI reduced from −0.23 (95% CI: −0.29, −0.17) in 1990 to −0.38 (95% CI: −0.44, −0.31) in 2021, indicating that, over time, the stroke burden became disproportionately concentrated in lower-SDI countries. Among the three stroke subtypes, ICH showed the highest SII (per 100,000 population) in 2021 at −344.6 (95% CI: −383.7, −305.5). The relative inequality for all three subtypes has clearly increased to varying degrees. In 2021, the CI for SAH was −0.40 (95% CI: −0.47, −0.32), reflecting a 62.5% increase compared to 1990. This increase indicated substantial health inequality for SAH in low-income countries.

### 3.6 Predictive analysis

**Figure 6:**
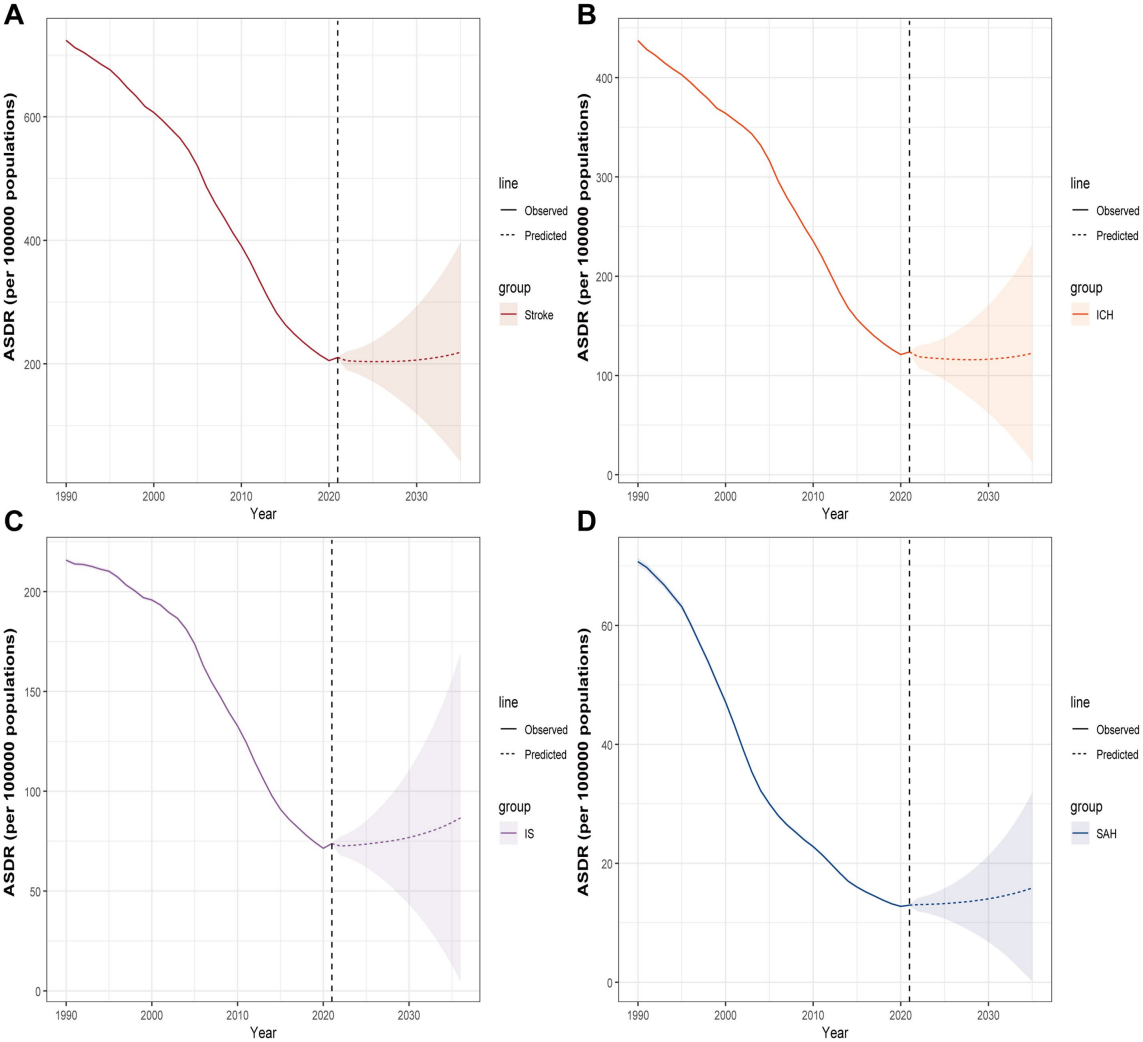
Projected ASDR for Stroke **(A)**, ICH **(B)**, IS **(C)**, and SAH **(D)** attributable to HAP by 2035 on the BAPC model; ASDR, age-standardized DALYs rate; ICH, intracerebral hemorrhage; IS, ischemic stroke; SAH, subarachnoid hemorrhage; HAP, household air pollution from solid fuels; BAPC, Bayesian age-period-cohort.

We employed the BAPC predictive model to forecast the HAP-related burden of stroke through 2035. The results indicated that the stroke burden would initially decrease before increasing, with the ASDR projected to reach 219.38 per 100,000 by 2035, reflecting a 4.29% increase compared to 2021 (Supplementary Table S6; Figure 6). The BAPC projections indicated an increasing trend across the three subtypes. IS exhibited the highest ASDR increase (12.07%), reaching 83.91 per 100,000 in 2035. The sensitivity analyses demonstrated that the overall trends of stroke and its three subtypes remained largely consistent with the primary projections described above (Supplementary Figures S3, S4).

## 4 Discussion

This study revealed that the global HAP-related stroke burden had decreased between 1990 and 2021, but it remained higher in men than in women and showed an age-related increase. The burden exhibited a polarized distribution across SDI levels, with lower SDI regions bearing the heaviest load. Decomposition analysis further demonstrated that population growth was the main contributor to changes in stroke burden in low and low-middle SDI regions, resulting in an absolute increase. Cross-national inequality analysis indicated that, between 1990 and 2021, relative inequalities in the global stroke burden have increased, with a disproportionate concentration of burden in countries with lower SDI, particularly in the SAH subtype, where significant health inequities persist. Predictive results suggested that, until 2035, the global stroke burden would initially decline before rising again, indicating that HAP-related stroke will continue to affect global health. Targeted interventions should be implemented promptly to mitigate the burden.

In 2021, premature deaths and DALYs attributable to HAP accounted for 38.5 and 47.2% of the total air pollution burden, respectively (4). In recent years, the HAP burden has decreased slightly due to greater use of clean fuels and improved sanitation (21, 22). However, in less developed regions, most residents still depend on traditional solid fuels to meet household energy needs (9). Several mechanisms may underlie the link between HAP and stroke. Indoor combustion of solid fuels releases harmful substances, such as particulate matter and black carbon, which can trigger inflammation, oxidative stress, and thrombosis, and may impair cardiometabolic function (23, 24). Additionally, PM_2.5_ from HAP can cross the blood–brain barrier, reach the central nervous system, and induce neurotoxic effects (25, 26). Stroke is a common consequence of hypertension, and prolonged exposure to HAP is associated with increased blood pressure (7). Furthermore, exposure to HAP elevates the likelihood of atrial fibrillation, which further promotes thromboembolic events, ultimately leading to IS (24, 27).

The widespread adoption of clean fuels and improved healthcare resource allocation have contributed to a substantial disparity in HAP-induced stroke burden across SDI areas, as the decline in high SDI areas exceeds that in low SDI areas by more than tenfold (4, 28, 29). Stroke burden was concentrated in South Asia, East Asia, and Africa, with South Asia recording the highest number of deaths and DALYs from HAP-attributable stroke in 2021 (10). In South Asia, HAP accounts for approximately 30% of PM_2.5_ in the environment, while this proportion is only 7% in high-income North America (10). Furthermore, nearly half of South Asia’s population is exposed to solid fuels, with over two-thirds of rural households using them for cooking, positioning HAP as the second-largest risk factor for CVD in rural areas (12). Developed countries have strict policies and regulations to control HAP, along with scientific health education on its impacts (9, 22). India and China ranked among the highest for HAP-attributable stroke deaths and DALYs, while Mozambique and the Solomon Islands reported the greatest ASRs. China and India’s heavy reliance on solid fuels and large rural populations pose significant challenges in managing indoor air pollution (8). Following the implementation of China’s Improved Stove Program and the Action Plan of Air Pollution Prevention and Control (APAPPC) (30, 31), along with India’s promotion of liquefied petroleum gas and clean energy subsidies for low-income households (32), the HAP-related stroke burden has declined in both countries in recent years. Mozambique had a significantly higher HAP-related stroke burden than other countries, with an increasing trend. Located in southeastern Africa, Mozambique ranks among the world’s poorest nations and has extremely low electrification coverage, compelling residents to depend largely on polluting fuels for lighting (33). In low-income countries, limited development provides few alternatives to solid fuels such as firewood, animal dung, and crop residues (12, 34). Therefore, targeted policies should be urgently implemented to improve energy access in low-income countries. As exemplified by the initiatives in China and India described above, community-level clean-stove programs and targeted subsidy policies implemented by governments can serve as effective strategies to reduce HAP burden in these settings. Additionally, health education to raise awareness of HAP and intervention strategies should be promoted.

This study found that the HAP-related stroke burden generally increased with age. This may be due to older adults spending more time indoors, either due to limited mobility or social role transitions, such as retirement, which leads to prolonged exposure to HAP (35, 36). Aging weakens the body’s antioxidant defense systems, including glutathione and superoxide dismutase, impairing the effective clearance of HAP-induced oxidative stress and inflammation, thus accelerating cerebrovascular damage (37, 38). Stroke prevalence also increases with age among older adults, who tend to have relatively low awareness of preventive healthcare. Consistent with previous studies, the HAP-related stroke burden was greater in males than in females (28, 36). The prevalence of underlying conditions such as hypertension and diabetes is typically higher in males, and these conditions may interact with HAP to further increase the risk of stroke (39, 40). Research has indicated that women consistently benefit more than men from both primary and secondary cardiovascular disease prevention (41). Although women are exposed to kitchen pollutants for longer periods due to frequent cooking (42, 43), men are more likely to encounter additional stroke-associated risk factors, like tobacco and alcohol consumption (4), potentially influencing the relationship between HAP and stroke.

This research found that we observed a steady increase in the contribution of IS in the global HAP-related stroke burden, while the other two subtypes showed varying degrees of decline, particularly in regions with higher SDI. The China-PAR study demonstrates that each 10 μg/m^3^ elevation in PM_2.5_ exposure correlates with elevated risks of ischemic stroke (20%) and hemorrhagic stroke (12%) (6). Prolonged PM_2.5_ exposure can activate platelets and trigger a systemic inflammatory response, accelerating atherosclerosis progression and increasing IS risk (6, 35). The mechanism of hemorrhagic stroke from PM_2.5_ exposure is closely linked to arterial vasoconstriction and elevated blood pressure (44). As global awareness of hypertension’s health risks has substantially grown, higher screening rates and standardized antihypertensive use have directly reduced the risk of hemorrhagic stroke. With the accelerated aging of populations in developed countries and the age-related rise in atherosclerosis and atrial fibrillation, IS has emerged as the dominant contributor in high-income settings (27, 45–47).

Despite recent reductions in the global HAP-attributable stroke burden, much of the epidemiological improvement has been offset by population growth and aging (48). In fact, population growth has consistently been the leading contributor to the rising stroke burden worldwide and across various SDI regions. This is consistent with recent research indicating that population growth significantly drives the burden of non-communicable diseases, including stroke, in lower SDI regions. Epidemiological changes have markedly reduced HAP-related stroke burden in middle SDI regions. First, these regions had the highest baseline values, reflecting the greatest initial burden. Second, countries in middle SDI regions, such as India and Bangladesh, have implemented measures to control indoor air pollution over the past three decades (22, 32, 49). Future global public health strategies should prioritize controlling HAP in underdeveloped regions. Countries should collaborate with the WHO and account for demographic changes when implementing public health interventions.

Analysis of health inequality showed that, compared with 1990, absolute health inequality in the burden of HAP-related stroke had decreased by 2021. However, relative inequality widened, with the burden increasingly concentrated in impoverished and underdeveloped regions reliant on solid fuels and lacking sufficient healthcare resources. Although global health has improved over the past three decades, the benefits have been concentrated in more developed countries (28). Improved medical services, enhanced stroke care, and proactive management of stroke risk factors in developed countries have significantly reduced the stroke burden. In 2021, the United States had 36.1 physicians per 10,000 people, whereas Papua New Guinea had fewer than one per 10,000 people (50). In low-income countries, only 13% of communities have access to the four most frequently used antihypertensive medications, and 31% of households cannot afford more than one antihypertensive drug (51). In underdeveloped regions, fuel collection in households reliant on polluting energy sources is often the responsibility of women and children, consuming significant time and limiting children’s access to education and women’s opportunities to improve household livelihoods (52). This perpetuates socioeconomic and health inequalities linked to energy poverty. In line with recent health inequality analyses, SAH demonstrates pronounced health disparities in low-income countries (53). Advances in neuroimaging technology in developed countries have enabled more rapid and accurate detection of SAH, thereby facilitating timely treatment and care (54). In low-income countries, the limited availability of MRI/CT and the shortage of neurologists pose significant challenges for aneurysm screening. Therefore, high-income regions could expand the coverage of basic healthcare resources in lower-income countries by providing increased medical assistance and health guidance (53). Additionally, governments in low SDI regions should adapt health policies to local needs to reduce heavy dependence on solid fuels, prioritizing access to clean fuels or improving stoves for households below the poverty line. As advocated by the WHO, global efforts should prioritize meeting the SDG target of ensuring clean cooking fuel accessibility for all by 2030. Furthermore, health education on cardiovascular diseases should be implemented in high-burden areas to raise awareness of self-management practices.

Based on the most recent GBD 2021 estimates, our research provided the first in-depth evaluation of HAP-attributable stroke burden at the global, regional, and national levels from 1990 to 2021. We employed advanced analytical methods—including decomposition analysis, health inequality analysis, and BAPC model-based predictions of disease burden trends. However, our research had several limitations. Firstly, owing to the absence of comprehensive monitoring systems and insufficient healthcare resources, some low SDI regions experience data gaps or possess only partial datasets. Although the GBD study employs advanced frameworks and the DisMod-MR tool to improve data accuracy and comparability, limitations in the quality of the original data persist (2, 4). Secondly, the GBD estimates predominantly emphasize disentangling the independent effects of HAP. In real-world settings, exposures to multiple risk factors such as indoor smoking and ambient PM_2.5_ often occur simultaneously and may interact synergistically (55). Thirdly, the GBD classified stroke into only three subtypes (IS, ICH, and SAH), excluding other types (e.g., silent strokes and strokes from specific causes), potentially leading to an underestimation of the stroke burden. Despite these limitations, our research highlights stark disparities in HAP-related stroke burden across regions with varying development levels, offering actionable insights for shaping targeted health policies and optimizing resource allocation to mitigate global health inequities.

## 5 Conclusion

In summary, the HAP-attributable stroke burden continues to pose a significant challenge to global public health. Although the overall burden has declined, men, older adults, and less developed regions continue to face a higher disease burden. Between 1990 and 2021, ICH represented the largest portion of the stroke burden, whereas the rising burden of IS requires attention. The relative inequity in the HAP-related stroke and its subtypes burden has increased over time, with a disproportionate concentration in countries with lower SDI (e.g., Zimbabwe and Lesotho), particularly for the SAH subtype.

## Data Availability

The data employed in this study were sourced from publicly available sources: the Institute for Health Metrics and Evaluation (IHME), accessible at: https://vizhub.healthdata.org/gbd-results/.

## Footnotes

^1^ https://vizhub.healthdata.org/gbd-results/
