# Supplementary figures and images for: Global burden, trends and health inequalities of stroke attributable to household air pollution, 1990–2021: a decomposition and prediction analysis
Source: Front Public Health. 2025 Sep 11;13:1625842. doi: 10.3389/fpubh.2025.1625842 (PMC12460407; doi:10.3389/fpubh.2025.1625842)

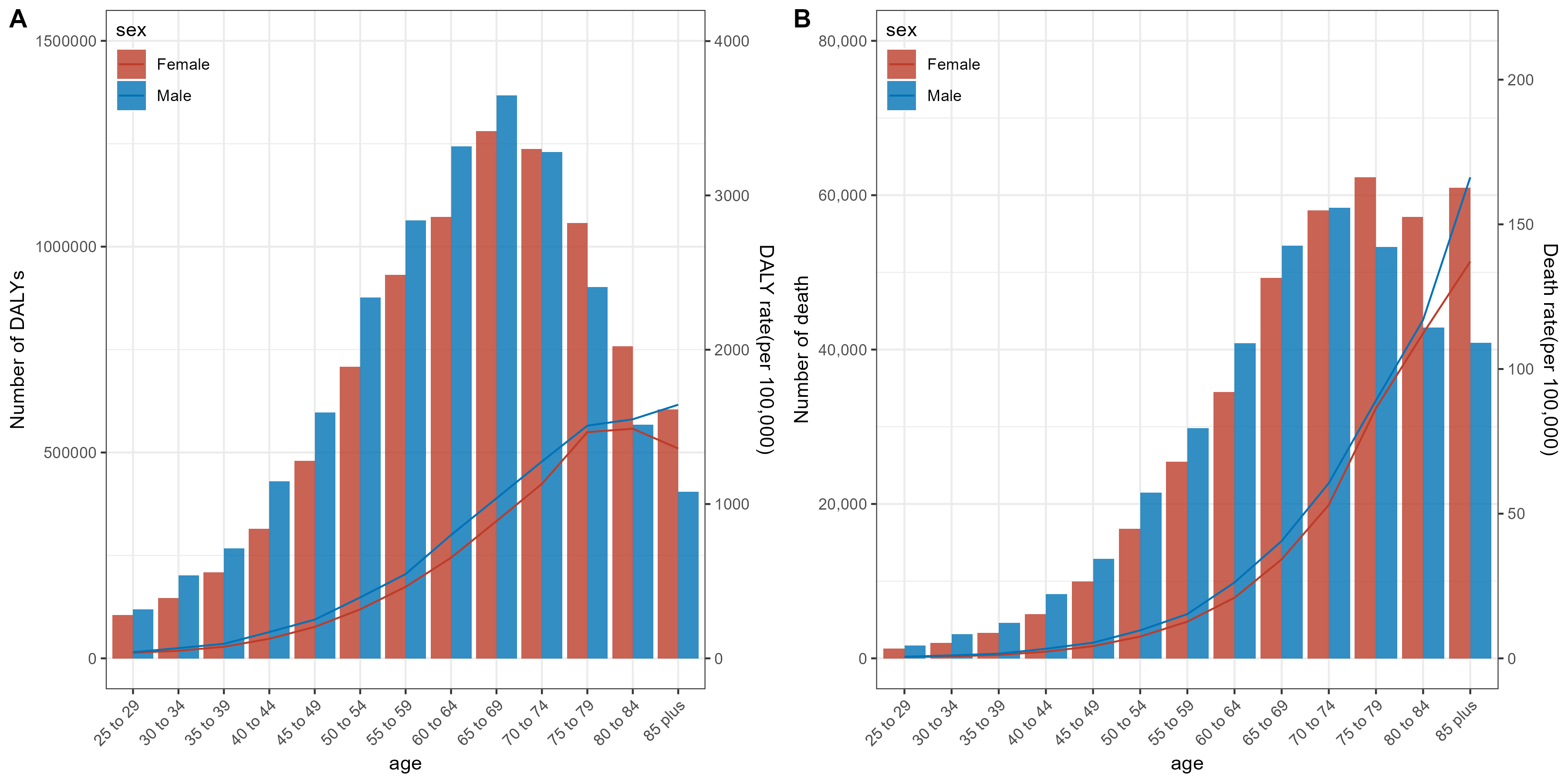

Supplement: Supplementary file 1 [file Image_1.tiff]

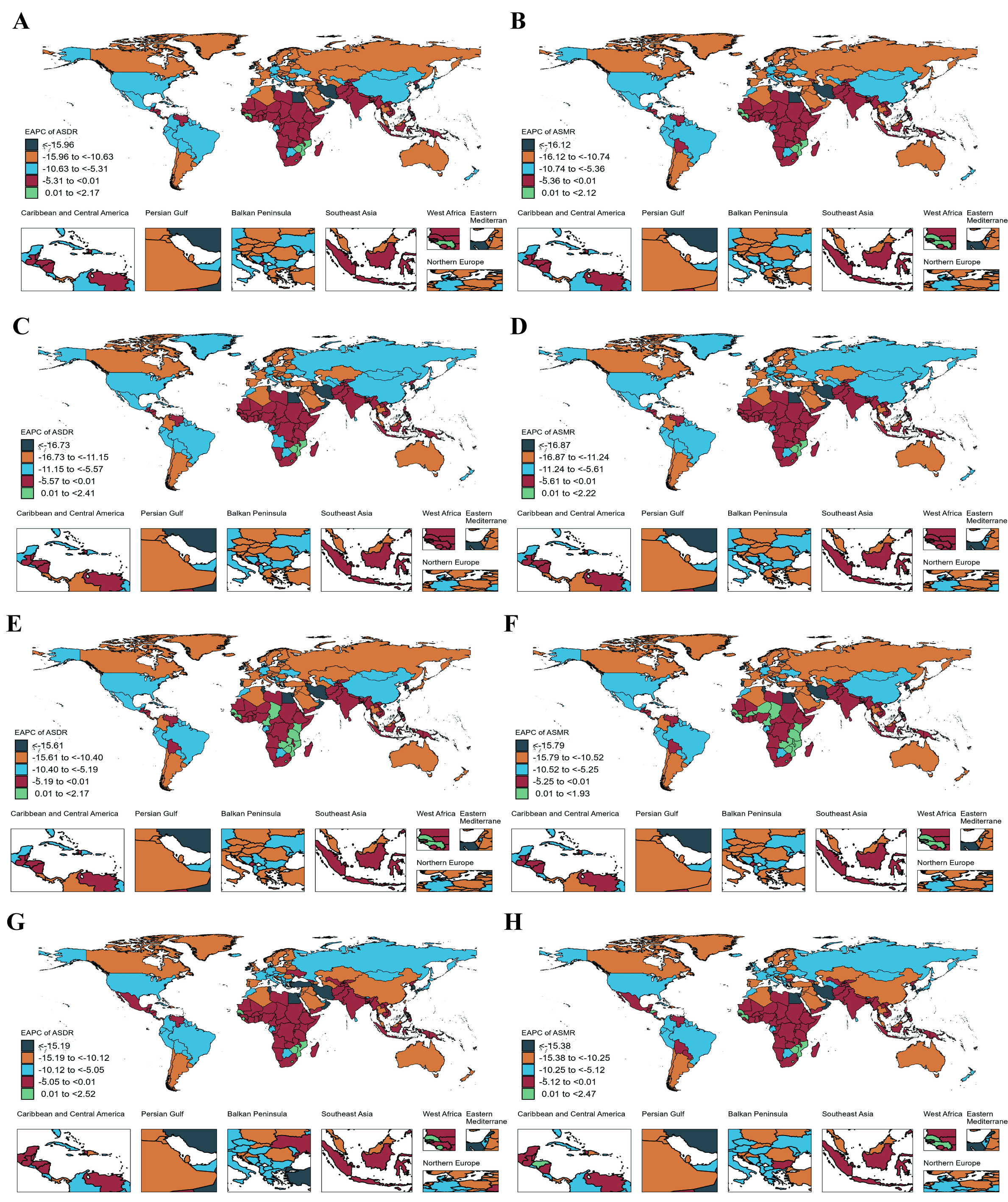

Supplement: Supplementary file 2 [file Image_2.tiff]

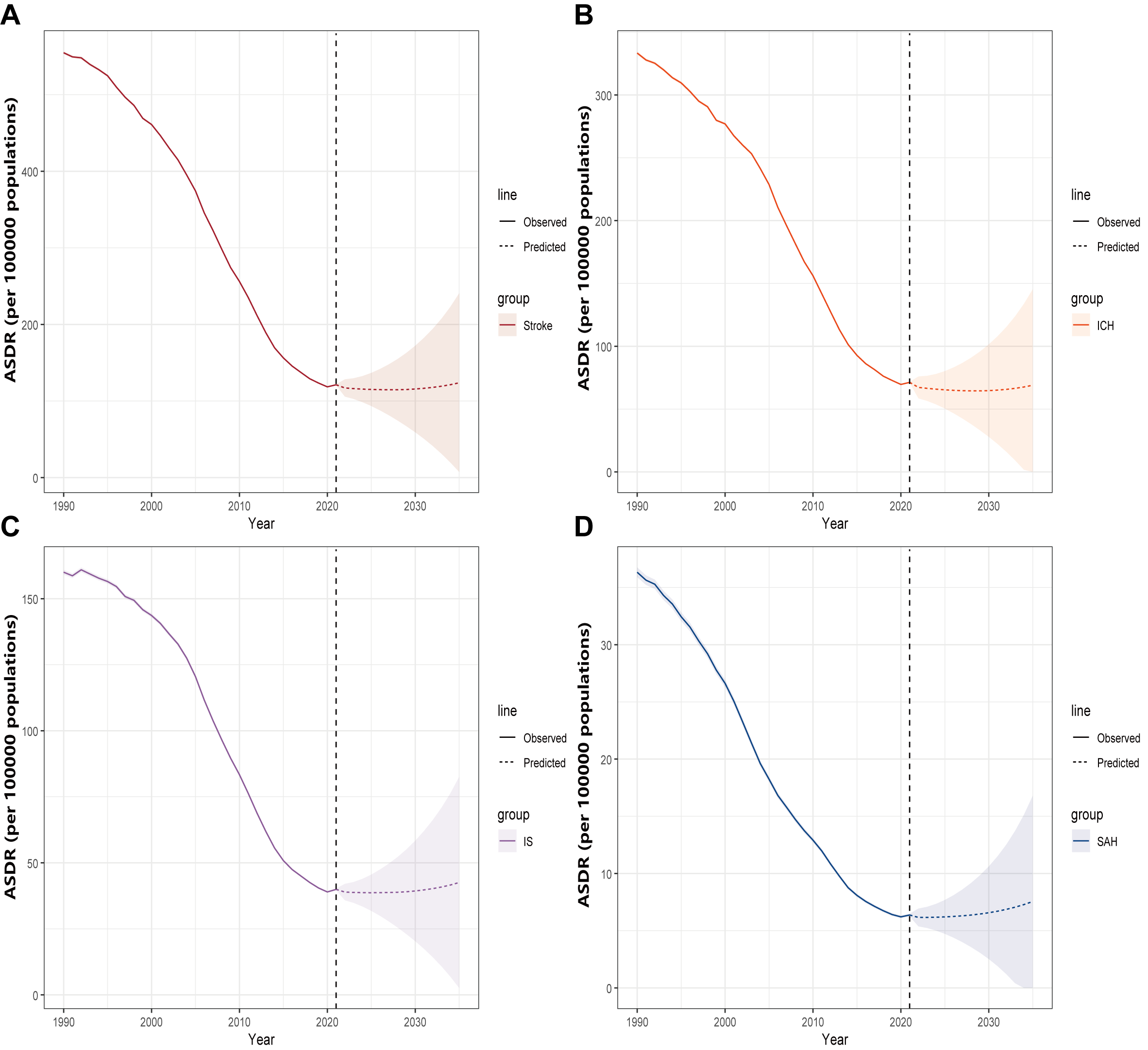

Supplement: Supplementary file 3 [file Image_3.tiff]

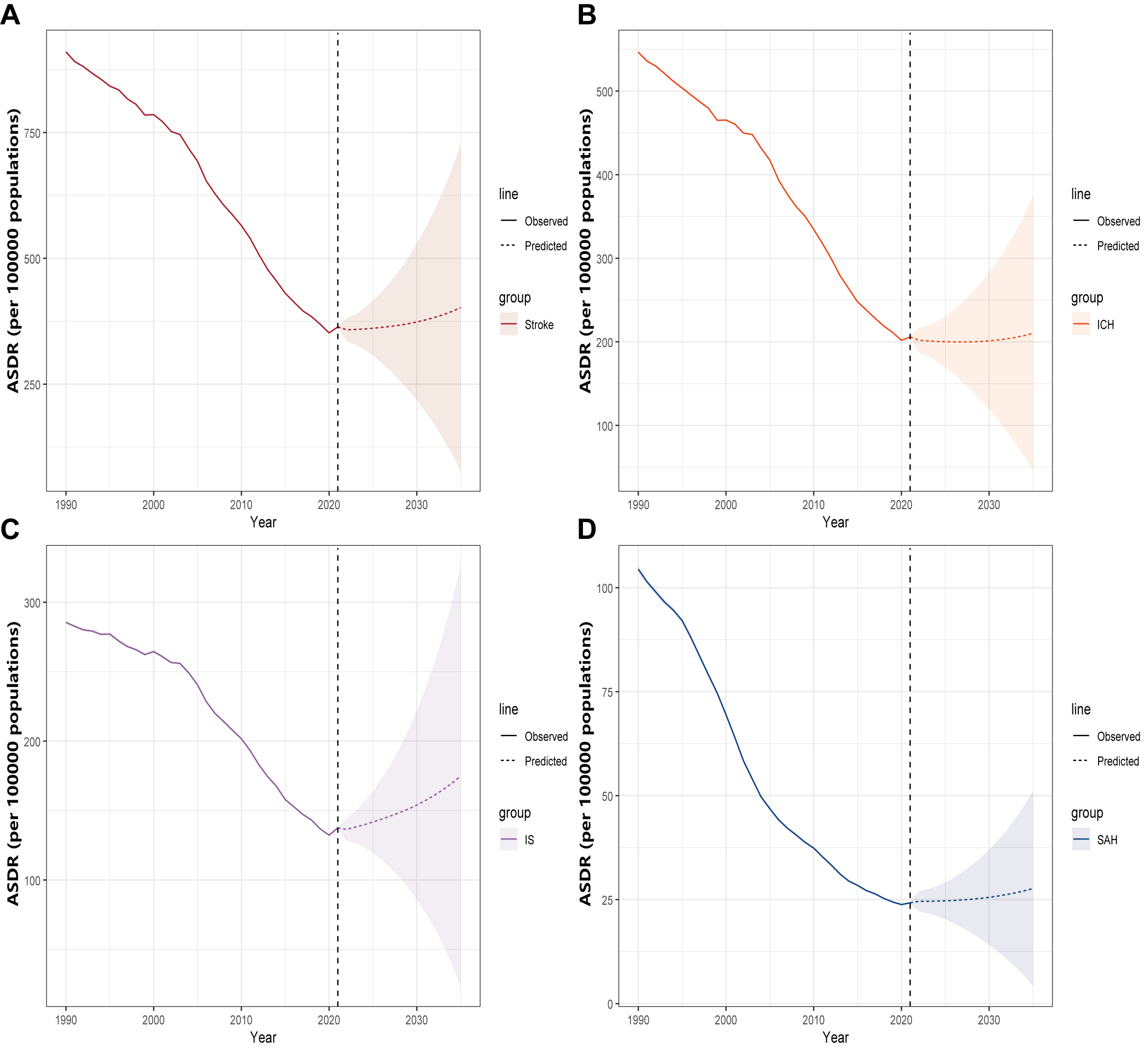

Supplement: Supplementary file 4 [file Image_4.tiff]
