# Supplementary material for: Global burden, trends and health inequalities of stroke attributable to household air pollution, 1990–2021: a decomposition and prediction analysis
Source: Front Public Health. 2025 Sep 11;13:1625842. doi: 10.3389/fpubh.2025.1625842 (PMC12460407; doi:10.3389/fpubh.2025.1625842)
Supplement: Supplementary file 5 [file Table_1.docx]

| **Supplementary Table 1. Global burden of stroke attributable to HAP in 1990 and 2021, and the temporal trends from 1990 to 2021.** | | | | | | | | | | |
| --- | --- | --- | --- | --- | --- | --- | --- | --- | --- | --- |
| **Location** | **1990** | | | | **2021** | | | | **EAPC (1990-2021)** | |
|  | **DALYs**  **NO. ×10^3^ (95% UI)** | **ASDR**  **NO. ×10^-5^ (95% UI)** | **Deaths**  **NO. ×10^3^ (95% UI)** | **ASMR**  **NO. ×10^-5^ (95% UI)** | **DALYs**  **NO. ×10^3^ (95% UI)** | **ASDR**  **NO. ×10^-5^ (95% UI)** | **Deaths**  **NO. ×10^3^ (95% UI)** | **ASMR**  **NO. ×10^-5^ (95% UI)** | **ASDR**  **NO. (95%CI)** | **ASMR**  **NO. (95%CI)** |
| Global | 28963.11 (22528.49to36117.91) | 723.74 (562.98to904.58) | 1160.31 (903.18to1454.29) | 31.43 (24.47to39.5) | 18175.21 (10672to30875.85) | 210.35 (123.22to358.01) | 758.48 (432.75to1311.61) | 8.95 (5.08to15.57) | -4.38 (-4.75 to -4) | -4.46 (-4.88 to -4.04) |
| **Sex** |  |  |  |  |  |  |  |  |  |  |
| Female | 14095.98 (11015.75to17824.18) | 658.18 (514.68to832.42) | 587.41 (459.3to746.49) | 28.53 (22.19to36.45) | 8906.7 (5217.4to14810.57) | 194.85 (114.43to323.26) | 386.89 (219.02to660.8) | 8.34 (4.73to14.22) | -4.3 (-4.64 to -3.96) | -4.36 (-4.74 to -3.97) |
| Male | 14867.12 (11236.22to18474.87) | 803.07 (604to1000.26) | 572.9 (429.07to717.74) | 35.3 (26.55to44.24) | 9268.51 (5307.92to16474.05) | 227.29 (129.25to407.06) | 371.59 (205.39to682.42) | 9.67 (5.28to17.98) | -4.48 (-4.89 to -4.06) | -4.61 (-5.08 to -4.14) |
| **Subtype** |  |  |  |  |  |  |  |  |  |  |
| Intracerebral hemorrhage | 17864.18 (13880.66to21806.85) | 437.28 (340.54to533.74) | 688.91 (538.09to842.49) | 18.05 (14.14to22.09) | 10761.86 (6305.26to17884.17) | 123.61 (72.34to205.7) | 421.3 (237.6to718.02) | 4.9 (2.75to8.38) | -4.42 (-4.82 to -4.02) | -4.57 (-5.03 to -4.12) |
| Ischemic stroke | 8144.12 (6138.2to10702.88) | 215.73 (162.27to282.62) | 368.14 (273.8to486.64) | 10.71 (7.96to14.26) | 6293.71 (3469.05to11655.62) | 73.78 (40.65to136.68) | 302.33 (163.07to560.67) | 3.64 (1.96to6.78) | -3.88 (-4.3 to -3.47) | -3.91 (-4.35 to -3.46) |
| Subarachnoid hemorrhage | 2954.81 (1918.54to4030.11) | 70.73 (45.12to96.73) | 103.26 (61.48to142.56) | 2.66 (1.57to3.68) | 1119.63 (575.79to1959.22) | 12.96 (6.67to22.68) | 34.85 (16.85to61.35) | 0.41 (0.2to0.72) | -6.12 (-6.34 to -5.9) | -6.83 (-7.11 to -6.56) |
| **SDI Region** |  |  |  |  |  |  |  |  |  |  |
| High SDI | 385.53 (136.95to879.66) | 34.97 (12.44to79.83) | 18.2 (6.29to41.75) | 1.61 (0.56to3.71) | 7.42 (0.01to73.15) | 0.35 (0to3.45) | 0.41 (0to4.06) | 0.02 (0to0.17) | -15.65 (-16.3 to -14.99) | -15.48 (-16.13 to -14.82) |
| High-middle SDI | 5847.46 (4080.8to8098.65) | 596.42 (416.46to828.84) | 248.07 (171.01to349.71) | 27.36 (18.89to39.11) | 626.89 (37.28to3288.77) | 31.83 (1.88to167.21) | 30.24 (1.73to162.24) | 1.54 (0.09to8.32) | -10.25 (-11.41 to -9.07) | -10.16 (-11.35 to -8.94) |
| Middle SDI | 12367.83 (9601.63to15297.11) | 1240.64 (966.93to1534.55) | 502.68 (390.1to626.21) | 59.67 (46.58to74.27) | 4520 (1097.73to11102.78) | 173.18 (41.6to425.77) | 205.88 (48.62to505.5) | 8.6 (2to21.15) | -6.56 (-7.26 to -5.86) | -6.47 (-7.21 to -5.73) |
| Low-middle SDI | 7112.9 (5679.8to8480.91) | 1162.09 (930.34to1389.09) | 269.56 (215.07to322.24) | 51.8 (41.35to62.04) | 8206.28 (5344.16to11525.42) | 575.61 (374.99to808.38) | 336.68 (218.68to472.61) | 26.61 (17.29to37.2) | -2.39 (-2.6 to -2.18) | -2.29 (-2.49 to -2.08) |
| Low SDI | 3227.99 (2621.68to3843.13) | 1437.86 (1172.33to1710.97) | 120.91 (98.58to144.45) | 64.51 (52.2to77.21) | 4798.95 (3778.92to5774.13) | 953.74 (747.82to1144.44) | 184.66 (145.44to223.14) | 44.12 (34.66to53.28) | -1.51 (-1.58 to -1.44) | -1.36 (-1.42 to -1.3) |
| **GBD Region** |  |  |  |  |  |  |  |  |  |  |
| Central Asia | 208.28 (90.26to397.49) | 451.75 (195.49to862.46) | 8.79 (3.84to16.69) | 20.78 (9to39.35) | 90.27 (37.48to202.06) | 114.1 (46.63to260.49) | 3.7 (1.49to8.62) | 5.39 (2.14to12.82) | -5.95 (-7.11 to -4.79) | -5.85 (-7.03 to -4.65) |
| Central Europe | 402.82 (102.54to1086.56) | 283.78 (72.44to761.94) | 20.46 (5.11to54.22) | 15.53 (3.92to40.97) | 45.57 (1.52to291.86) | 19.88 (0.66to127.61) | 2.72 (0.09to17.47) | 1.13 (0.04to7.25) | -10.05 (-10.86 to -9.22) | -9.87 (-10.65 to -9.08) |
| Eastern Europe | 154.95 (33.69to599.76) | 58.24 (12.55to225.68) | 7.73 (1.57to30.67) | 3.14 (0.62to12.56) | 30.01 (4.33to131.98) | 8.67 (1.25to37.97) | 1.54 (0.21to6.95) | 0.43 (0.06to1.95) | -8.7 (-10.33 to -7.04) | -8.98 (-10.62 to -7.31) |
| Australasia | 0.18 (0to2) | 0.78 (0to8.77) | 0.01 (0to0.11) | 0.05 (0to0.51) | 0.01 (0to0.07) | 0.02 (0to0.12) | 0 (0to0) | 0 (0to0.01) | -12.36 (-13.08 to -11.62) | -12.67 (-13.35 to -11.99) |
| High-income Asia Pacific | 10.74 (0.91to52.84) | 5.42 (0.45to26.77) | 0.46 (0.04to2.3) | 0.25 (0.02to1.24) | 0.17 (0to1.21) | 0.03 (0to0.24) | 0.01 (0to0.07) | 0 (0to0.01) | -14.96 (-16.21 to -13.7) | -15.09 (-16.29 to -13.88) |
| High-income North America | 0.24 (0to1.91) | 0.07 (0to0.54) | 0.01 (0to0.1) | 0 (0to0.03) | 0.05 (0to0.26) | 0.01 (0to0.04) | 0 (0to0.01) | 0 (0to0) | -7.99 (-8.35 to -7.62) | -8.35 (-8.76 to -7.94) |
| Western Europe | 9.49 (0.11to74.27) | 1.61 (0.02to12.57) | 0.57 (0.01to4.48) | 0.09 (0to0.74) | 0.26 (0to2.27) | 0.03 (0to0.22) | 0.02 (0to0.16) | 0 (0to0.01) | -13.09 (-13.76 to -12.42) | -13.2 (-13.86 to -12.53) |
| Andean Latin America | 68.44 (32.52to109.43) | 319.03 (151.14to507.99) | 2.68 (1.27to4.26) | 14.04 (6.61to22.33) | 20.06 (3.53to61.64) | 33.49 (5.91to103) | 0.86 (0.15to2.66) | 1.49 (0.27to4.64) | -7.4 (-7.81 to -6.99) | -7.35 (-7.77 to -6.93) |
| Caribbean | 123.61 (87.83to163.62) | 467.31 (331.65to619.29) | 4.9 (3.45to6.52) | 19.64 (13.63to26.24) | 134.55 (93.06to186.15) | 253.46 (175.4to350.3) | 5.21 (3.61to7.15) | 9.73 (6.74to13.35) | -1.89 (-2.07 to -1.7) | -2.13 (-2.3 to -1.97) |
| Central Latin America | 121.53 (54.97to222.22) | 143.75 (63.92to265.13) | 4.94 (2.17to9.2) | 6.83 (2.96to12.89) | 87.31 (34.56to193.02) | 34.98 (13.85to77.42) | 3.85 (1.5to8.46) | 1.61 (0.63to3.55) | -4.79 (-4.88 to -4.69) | -4.83 (-4.94 to -4.72) |
| Southern Latin America | 50.98 (12.69to130.03) | 112.4 (27.96to286.48) | 2.27 (0.57to5.7) | 5.3 (1.32to13.3) | 2.27 (0.01to20.93) | 2.6 (0.01to23.97) | 0.11 (0to1.02) | 0.12 (0to1.13) | -12.05 (-12.31 to -11.79) | -11.94 (-12.19 to -11.7) |
| Tropical Latin America | 319.67 (158.1to560.16) | 354.1 (176.82to614.81) | 13.19 (6.64to22.8) | 17.36 (8.75to29.23) | 60.66 (12.65to171.51) | 23.73 (4.95to67.07) | 2.77 (0.58to7.69) | 1.12 (0.23to3.1) | -8.81 (-9.13 to -8.48) | -8.78 (-9.1 to -8.47) |
| North Africa and Middle East | 631.88 (415.68to995.07) | 385.48 (251.89to613.16) | 25.7 (16.74to40.96) | 18.63 (11.88to30.26) | 346.34 (233.51to496.62) | 73.54 (49.73to106.62) | 13.35 (8.96to19.48) | 3.37 (2.26to5.01) | -5.82 (-6 to -5.65) | -5.96 (-6.13 to -5.78) |
| Southeast Asia | 4020.22 (3069.79to4980.32) | 1582.98 (1206.91to1954.43) | 154.52 (117.22to190.79) | 72.28 (54.98to89.52) | 2832.23 (1192.82to5184.18) | 438.6 (184.71to800.66) | 116.01 (48.92to211.64) | 20.51 (8.66to37.52) | -4.08 (-4.56 to -3.6) | -4 (-4.5 to -3.49) |
| South Asia | 5578.43 (4429.77to6736.83) | 970.19 (769.98to1168.55) | 208.74 (165.95to250.71) | 43.36 (34.26to52.29) | 6578.49 (4297.47to9397.01) | 449.51 (293.73to643.41) | 271.52 (176.56to387.47) | 20.87 (13.55to29.81) | -2.6 (-2.78 to -2.42) | -2.49 (-2.66 to -2.31) |
| East Asia | 14253.05 (10975.26to17805.28) | 1767.26 (1364.5to2208.27) | 590.38 (454.07to742.39) | 89.68 (69.03to112.48) | 3581.77 (988.43to10149.21) | 168.85 (46.18to478.75) | 169.47 (44.53to488.28) | 8.51 (2.19to24.52) | -7.99 (-8.8 to -7.17) | -8.02 (-8.88 to -7.15) |
| Oceania | 56.32 (40.05to74.86) | 1874.09 (1345.77to2459.25) | 1.95 (1.4to2.55) | 82.54 (60.09to107.08) | 99.96 (69.42to134.21) | 1297.23 (905.22to1724.07) | 3.53 (2.44to4.7) | 57.75 (40.13to76.4) | -1.2 (-1.23 to -1.16) | -1.16 (-1.21 to -1.11) |
| Central Sub-Saharan Africa | 331.01 (241.02to430.26) | 1537.7 (1126.78to1989.48) | 12.18 (8.83to15.88) | 70.74 (50.77to90.95) | 545.76 (378.9to743.74) | 1064.02 (743.33to1429.11) | 20.76 (14.34to28.33) | 51.42 (35.68to71.69) | -1.4 (-1.53 to -1.28) | -1.23 (-1.36 to -1.11) |
| Eastern Sub-Saharan Africa | 1356.74 (1095.73to1638.51) | 1814.58 (1475.23to2173.53) | 50.31 (40.78to60.58) | 80.78 (65.85to97.03) | 1918.74 (1506.9to2323.25) | 1146.3 (904.36to1387.56) | 72.76 (57.13to88.65) | 52.98 (41.9to64.77) | -1.71 (-1.78 to -1.64) | -1.56 (-1.62 to -1.5) |
| Southern Sub-Saharan Africa | 115.38 (65.3to177.07) | 428.09 (244.5to649.46) | 4.52 (2.61to6.82) | 19.28 (11.22to29.02) | 148.11 (94.31to238.03) | 257.68 (162.45to421.58) | 5.9 (3.68to9.52) | 11.77 (6.98to19.71) | -1.68 (-2.32 to -1.04) | -1.64 (-2.35 to -0.93) |
| Western Sub-Saharan Africa | 1149.15 (854.22to1430.59) | 1339.59 (1000.23to1664.72) | 45.99 (34.01to57.31) | 62.9 (46.18to78.03) | 1652.62 (1136.35to2215.55) | 856.49 (589.6to1143.86) | 64.39 (44.7to85.65) | 40.88 (28.16to54.28) | -1.75 (-1.93 to -1.56) | -1.69 (-1.87 to -1.5) |
| HAP, household air pollution from solid fuels; DALYs, disability-adjusted life years; ASDR, age-standardized DALYs rate; ASMR, age-standardized deaths rate; EAPC, estimated annual percentage change; CI, confidential interval; UI, uncertainty interval | | | | | | | | | | |
