# Supplementary material for: Global burden, trends and health inequalities of stroke attributable to household air pollution, 1990–2021: a decomposition and prediction analysis
Source: Front Public Health. 2025 Sep 11;13:1625842. doi: 10.3389/fpubh.2025.1625842 (PMC12460407; doi:10.3389/fpubh.2025.1625842)
Supplement: Supplementary file 6 [file Table_2.docx]

| **Supplementary Table 2. Burden of three stroke subtypes attributable to HAP in 21 GBD regions in 1990 and 2021, and temporal trends from 1990 to 2021.** | | | | | | | | | | |
| --- | --- | --- | --- | --- | --- | --- | --- | --- | --- | --- |
| **Location** | **1990** | | | | **2021** | | | | **EAPC (1990-2021)** | |
|  | **DALYs**  **NO. ×10^3^ (95% UI)** | **ASDR**  **NO. ×10^-5^ (95% UI)** | **Deaths**  **NO. ×10^3^ (95% UI)** | **ASMR**  **NO. ×10^-5^ (95% UI)** | **DALYs**  **NO. ×10^3^ (95% UI)** | **ASDR**  **NO. ×10^-5^ (95% UI)** | **Deaths**  **NO. ×10^3^ (95% UI)** | **ASMR**  **NO. ×10^-5^ (95% UI)** | **ASDR**  **NO. (95%CI)** | **ASMR**  **NO. (95%CI)** |
| **Intracerebral hemorrhage** |  |  |  |  |  |  |  |  |  |  |
| Central Asia | 114.69 (51.65to207.97) | 241.28 (108.62to437.49) | 4.58 (2.07to8.25) | 10.41 (4.72to18.82) | 40.43 (16.29to92.65) | 48.08 (19.12to112.18) | 1.57 (0.61to3.72) | 2.11 (0.81to5.13) | -6.73 (-8.04 to -5.41) | -6.64 (-7.99 to -5.26) |
| Central Europe | 143.43 (37.26to389.02) | 97.28 (25.78to262.41) | 6.27 (1.68to16.72) | 4.46 (1.24to11.75) | 12.26 (0.43to76.88) | 5.61 (0.19to35.49) | 0.67 (0.03to4.1) | 0.29 (0.01to1.76) | -10.87 (-11.79 to -9.94) | -10.46 (-11.35 to -9.56) |
| Eastern Europe | 50.65 (17.18to157.66) | 18.23 (6.2to56.7) | 2.05 (0.7to6.42) | 0.76 (0.26to2.38) | 7.92 (1.31to32.35) | 2.4 (0.4to9.87) | 0.32 (0.05to1.31) | 0.09 (0.02to0.38) | -9.14 (-10.7 to -7.54) | -9.36 (-10.88 to -7.81) |
| Australasia | 0.05 (0to0.5) | 0.19 (0to2.13) | 0 (0to0.02) | 0.01 (0to0.1) | 0 (0to0.02) | 0.01 (0to0.03) | 0 (0to0) | 0 (0to0) | -11.91 (-12.65 to -11.17) | -11.68 (-12.37 to -10.99) |
| High-income Asia Pacific | 5.11 (0.47to23.92) | 2.52 (0.23to11.82) | 0.2 (0.02to0.95) | 0.1 (0.01to0.49) | 0.05 (0to0.36) | 0.01 (0to0.08) | 0 (0to0.02) | 0 (0to0) | -15.79 (-17.19 to -14.37) | -15.61 (-17.01 to -14.18) |
| High-income North America | 0.06 (0to0.49) | 0.02 (0to0.14) | 0 (0to0.02) | 0 (0to0.01) | 0.01 (0to0.08) | 0 (0to0.01) | 0 (0to0) | 0 (0to0) | -7.19 (-7.53 to -6.84) | -7.15 (-7.54 to -6.76) |
| Western Europe | 2.96 (0.03to23.22) | 0.52 (0.01to4.12) | 0.15 (0to1.14) | 0.02 (0to0.19) | 0.08 (0to0.71) | 0.01 (0to0.08) | 0 (0to0.04) | 0 (0to0) | -12.99 (-13.61 to -12.36) | -12.65 (-13.26 to -12.03) |
| Andean Latin America | 36.69 (17.22to60.38) | 165.11 (77.28to269.69) | 1.32 (0.61to2.14) | 6.55 (3.05to10.59) | 8.91 (1.59to26.95) | 14.65 (2.63to44.15) | 0.35 (0.06to1.05) | 0.6 (0.11to1.79) | -7.96 (-8.36 to -7.56) | -7.85 (-8.26 to -7.44) |
| Caribbean | 75.05 (52.79to98.68) | 279.01 (196.18to367.78) | 2.69 (1.9to3.54) | 10.37 (7.29to13.68) | 78.78 (52.22to111.06) | 148.38 (98.38to208.61) | 2.78 (1.84to3.85) | 5.2 (3.44to7.2) | -1.98 (-2.17 to -1.78) | -2.16 (-2.32 to -1.99) |
| Central Latin America | 60.23 (28.87to105.72) | 66.85 (31.62to118.04) | 2.2 (1.03to3.91) | 2.78 (1.3to4.96) | 41.65 (17.36to87.5) | 16.36 (6.82to34.38) | 1.69 (0.69to3.56) | 0.69 (0.28to1.45) | -4.88 (-4.98 to -4.77) | -4.79 (-4.91 to -4.67) |
| Southern Latin America | 22.93 (5.55to58.94) | 49.15 (11.91to126.41) | 0.88 (0.21to2.24) | 1.93 (0.47to4.91) | 0.86 (0to7.85) | 1.01 (0to9.19) | 0.04 (0to0.34) | 0.04 (0to0.38) | -12.5 (-12.77 to -12.23) | -12.28 (-12.54 to -12.02) |
| Tropical Latin America | 152.15 (74.4to267.94) | 151.55 (74.5to264.91) | 5.26 (2.6to9.1) | 5.88 (2.92to10.02) | 24.67 (5.11to68.66) | 9.42 (1.95to26.22) | 0.94 (0.2to2.61) | 0.37 (0.08to1.02) | -9.16 (-9.51 to -8.82) | -9.06 (-9.4 to -8.72) |
| North Africa and Middle East | 310.48 (207.06to479.5) | 173.1 (115.42to270.2) | 11.28 (7.5to17.67) | 7.3 (4.75to11.59) | 154.59 (97.46to230.72) | 29.69 (18.68to44.96) | 5.14 (3.17to7.84) | 1.15 (0.7to1.76) | -6.27 (-6.49 to -6.05) | -6.5 (-6.72 to -6.28) |
| Southeast Asia | 2705.48 (2070.13to3345.8) | 1002.75 (761.99to1242.79) | 98.18 (74.86to121.76) | 42.01 (31.98to52.26) | 1778.67 (751.4to3247.59) | 258.96 (109.47to474.97) | 67.29 (28.49to123.6) | 10.89 (4.62to19.86) | -4.33 (-4.8 to -3.85) | -4.32 (-4.81 to -3.82) |
| South Asia | 3464.42 (2629.87to4297.6) | 565.06 (427.19to701.84) | 122.92 (92.69to153.19) | 23 (17.33to28.77) | 3797.1 (2438.69to5462.22) | 247.18 (158.25to356.52) | 144.75 (91.46to209.28) | 10.27 (6.41to14.96) | -2.72 (-2.9 to -2.55) | -2.66 (-2.83 to -2.49) |
| East Asia | 8621.11 (6577.85to10951.41) | 1042.67 (800.98to1324.24) | 354.34 (271.39to451.98) | 52.09 (40.18to66.07) | 2013.1 (589.99to5460.88) | 94.02 (27.43to255.14) | 94.43 (26.05to261.23) | 4.65 (1.26to12.96) | -8.11 (-9 to -7.2) | -8.14 (-9.07 to -7.19) |
| Oceania | 43.18 (30.15to57.97) | 1377.64 (982.26to1821.1) | 1.5 (1.07to1.98) | 58.82 (42.13to77.4) | 75.96 (51.19to102.49) | 944.06 (642.96to1273.58) | 2.67 (1.81to3.6) | 40.69 (27.64to54.67) | -1.21 (-1.25 to -1.18) | -1.18 (-1.21 to -1.14) |
| Central Sub-Saharan Africa | 231.24 (161.37to306.58) | 979.4 (694.38to1289.19) | 8.26 (5.82to10.91) | 41.71 (29.65to55) | 368.39 (255.53to507.38) | 646.12 (451.72to886.79) | 13.38 (9.31to18.42) | 28.71 (20.07to39.39) | -1.54 (-1.68 to -1.4) | -1.39 (-1.53 to -1.24) |
| Eastern Sub-Saharan Africa | 1032.88 (809.08to1272.75) | 1304.13 (1027.74to1608.07) | 37.35 (29.4to46.11) | 54.8 (43.36to67.4) | 1315.62 (1012.68to1628.49) | 727.19 (565.3to895.38) | 47.88 (37.14to59.01) | 31.38 (24.3to38.72) | -2.15 (-2.23 to -2.06) | -2.04 (-2.11 to -1.96) |
| Southern Sub-Saharan Africa | 70.42 (40.01to107.9) | 243.79 (141.42to368.29) | 2.57 (1.51to3.83) | 10 (5.93to14.84) | 90.29 (58to141.67) | 146.15 (93.69to229.53) | 3.31 (2.13to5.19) | 5.94 (3.77to9.35) | -1.61 (-2.25 to -0.97) | -1.68 (-2.34 to -1.01) |
| Western Sub-Saharan Africa | 720.96 (511.99to931.13) | 784.95 (553.73to1013.95) | 26.93 (18.89to35.2) | 33.37 (23.05to44.18) | 952.52 (649.11to1289.27) | 445.2 (300.48to600.25) | 34.08 (22.96to45.93) | 18.9 (12.77to25.43) | -2.15 (-2.3 to -1.99) | -2.15 (-2.3 to -2) |
| **Ischemic stroke** |  |  |  |  |  |  |  |  |  |  |
| **Location** | **1990** | | | | **2021** | | | | **EAPC (1990-2021)** | |
|  | **DALYs**  **NO. ×10^3^ (95% UI)** | **ASDR**  **NO. ×10^-5^ (95% UI)** | **Deaths**  **NO. ×10^3^ (95% UI)** | **ASMR**  **NO. ×10^-5^ (95% UI)** | **DALYs**  **NO. ×10^3^ (95% UI)** | **ASDR**  **NO. ×10^-5^ (95% UI)** | **Deaths**  **NO. ×10^3^ (95% UI)** | **ASMR**  **NO. ×10^-5^ (95% UI)** | **ASDR**  **NO. (95%CI)** | **ASMR**  **NO. (95%CI)** |
| Central Asia | 84.98 (35.12to171.4) | 193.27 (79.9to390.23) | 3.95 (1.64to7.89) | 9.8 (4.05to19.66) | 43.72 (18.43to96.35) | 59.15 (24.45to133.33) | 1.94 (0.79to4.47) | 3.03 (1.21to7.1) | -5.34 (-6.37 to -4.3) | -5.31 (-6.35 to -4.25) |
| Central Europe | 237.65 (58.19to643.38) | 171.48 (41.91to462.07) | 13.54 (3.22to36.23) | 10.62 (2.53to28.32) | 31.33 (1.05to203.46) | 13.2 (0.45to85.71) | 1.98 (0.06to12.94) | 0.81 (0.03to5.28) | -9.64 (-10.41 to -8.87) | -9.65 (-10.39 to -8.9) |
| Eastern Europe | 98.38 (15.17to418.69) | 37.79 (5.87to160.66) | 5.47 (0.83to23.59) | 2.3 (0.35to9.89) | 20.51 (2.82to92.89) | 5.76 (0.8to25.95) | 1.16 (0.15to5.38) | 0.32 (0.04to1.49) | -8.61 (-10.25 to -6.93) | -8.93 (-10.6 to -7.24) |
| Australasia | 0.11 (0to1.25) | 0.49 (0to5.52) | 0.01 (0to0.08) | 0.03 (0to0.36) | 0.01 (0to0.04) | 0.01 (0to0.07) | 0 (0to0) | 0 (0to0) | -12.84 (-13.57 to -12.1) | -13.24 (-13.91 to -12.57) |
| High-income Asia Pacific | 4.32 (0.35to22.43) | 2.25 (0.18to11.94) | 0.22 (0.02to1.15) | 0.12 (0.01to0.67) | 0.09 (0to0.67) | 0.02 (0to0.11) | 0.01 (0to0.05) | 0 (0to0.01) | -14.88 (-16.01 to -13.73) | -15.09 (-16.16 to -14) |
| High-income North America | 0.15 (0to1.18) | 0.04 (0to0.32) | 0.01 (0to0.07) | 0 (0to0.02) | 0.03 (0to0.15) | 0 (0to0.02) | 0 (0to0.01) | 0 (0to0) | -8.54 (-8.96 to -8.11) | -9.07 (-9.55 to -8.58) |
| Western Europe | 6.1 (0.07to47.65) | 1 (0.01to7.77) | 0.41 (0to3.22) | 0.07 (0to0.53) | 0.16 (0to1.37) | 0.01 (0to0.12) | 0.01 (0to0.1) | 0 (0to0.01) | -13.51 (-14.22 to -12.79) | -13.67 (-14.36 to -12.97) |
| Andean Latin America | 19.7 (9.05to31.47) | 104.84 (48.19to167.09) | 1.02 (0.47to1.63) | 5.97 (2.75to9.51) | 6.83 (1.18to21.75) | 11.96 (2.06to38.1) | 0.37 (0.06to1.18) | 0.67 (0.11to2.14) | -7.14 (-7.55 to -6.73) | -7.19 (-7.61 to -6.77) |
| Caribbean | 36.61 (25.15to50.21) | 146.05 (99.36to201.54) | 1.87 (1.26to2.6) | 8.03 (5.39to11.27) | 39.87 (27.13to56.56) | 74.51 (50.77to105.6) | 1.98 (1.34to2.78) | 3.69 (2.49to5.18) | -2.05 (-2.2 to -1.89) | -2.29 (-2.45 to -2.13) |
| Central Latin America | 45.59 (18.43to89.05) | 61.63 (24.83to120.89) | 2.33 (0.94to4.57) | 3.59 (1.45to7.05) | 31.82 (11.93to73.97) | 13.32 (4.99to30.96) | 1.73 (0.65to3.95) | 0.75 (0.28to1.72) | -5.09 (-5.18 to -5) | -5.15 (-5.25 to -5.04) |
| Southern Latin America | 22.07 (5.78to53.94) | 50.47 (13.17to123.5) | 1.22 (0.32to2.99) | 2.99 (0.78to7.36) | 1.09 (0to10.06) | 1.21 (0to11.1) | 0.06 (0to0.58) | 0.07 (0to0.62) | -11.86 (-12.12 to -11.59) | -11.88 (-12.13 to -11.63) |
| Tropical Latin America | 132.19 (65.91to230.89) | 171.53 (87.01to294.32) | 7.03 (3.57to11.99) | 10.6 (5.37to17.7) | 26.58 (5.49to76.5) | 10.74 (2.22to30.86) | 1.54 (0.32to4.3) | 0.64 (0.13to1.78) | -8.85 (-9.15 to -8.55) | -8.85 (-9.16 to -8.54) |
| North Africa and Middle East | 289.46 (181.18to475.78) | 195.46 (120.94to324.61) | 13.41 (8.2to22.24) | 10.7 (6.48to17.94) | 173.8 (113.14to257.99) | 40.55 (26.55to60.07) | 7.69 (5.02to11.51) | 2.11 (1.37to3.18) | -5.46 (-5.61 to -5.32) | -5.62 (-5.77 to -5.46) |
| Southeast Asia | 1056.3 (809.44to1313.94) | 491.96 (376.21to611.09) | 48.64 (36.73to60.48) | 27.13 (20.55to33.65) | 914.42 (377.47to1718.07) | 160.16 (66.25to299.47) | 44.57 (18.79to82.58) | 8.96 (3.79to16.56) | -3.54 (-4.04 to -3.04) | -3.46 (-3.99 to -2.93) |
| South Asia | 1569.21 (1183.66to2059.25) | 325.73 (246.7to421.45) | 70.03 (52.86to91.78) | 17.7 (13.5to22.85) | 2225.46 (1432.34to3276.12) | 168.39 (108.2to246.62) | 109.89 (70.26to160.57) | 9.48 (6.07to13.83) | -2.35 (-2.54 to -2.16) | -2.23 (-2.42 to -2.05) |
| East Asia | 3743.79 (2813.66to4755.56) | 506.2 (380.38to641.47) | 163.77 (122.11to208.25) | 27.49 (20.76to34.91) | 1398.15 (346.67to4212.82) | 66.82 (16.45to201.58) | 68.19 (16.13to206.69) | 3.52 (0.82to10.7) | -6.96 (-7.78 to -6.12) | -7.07 (-7.95 to -6.18) |
| Oceania | 8.1 (5.64to10.75) | 358.09 (249.47to474.27) | 0.31 (0.21to0.43) | 18.92 (13to25.23) | 15.58 (10.83to21.64) | 264.91 (185.17to366.07) | 0.64 (0.44to0.91) | 14.11 (9.6to20.07) | -1.04 (-1.12 to -0.96) | -1.01 (-1.11 to -0.9) |
| Central Sub-Saharan Africa | 87.83 (64.41to116.7) | 513.27 (372.4to670.52) | 3.59 (2.54to4.78) | 27.54 (19.58to36.34) | 153.64 (104.42to213.28) | 383.05 (262.02to543.3) | 6.74 (4.43to9.82) | 21.55 (14.1to31.6) | -1.19 (-1.3 to -1.08) | -1.03 (-1.15 to -0.92) |
| Eastern Sub-Saharan Africa | 269.63 (208.19to346.21) | 449.34 (350.54to576.02) | 11.44 (8.72to15.07) | 23.98 (18.53to31.54) | 518.69 (404.66to650.78) | 379.93 (297.87to472.62) | 22.66 (17.51to28.32) | 20.36 (15.59to25.46) | -0.68 (-0.73 to -0.63) | -0.65 (-0.71 to -0.6) |
| Southern Sub-Saharan Africa | 40.92 (22.88to63.1) | 171.18 (95.45to262.87) | 1.84 (1.05to2.83) | 8.87 (5.03to13.62) | 50.68 (30.39to88.41) | 101 (58.75to179.37) | 2.39 (1.37to4.19) | 5.5 (3.01to9.91) | -1.89 (-2.55 to -1.22) | -1.67 (-2.43 to -0.9) |
| Western Sub-Saharan Africa | 391.02 (276.91to521.05) | 518.32 (360.4to694.34) | 18.03 (12.32to24.65) | 28.37 (19.57to38.82) | 641.26 (446.2to889.25) | 388.09 (270.16to535.91) | 28.79 (19.97to39.75) | 21.26 (14.63to29.4) | -1.21 (-1.44 to -0.98) | -1.2 (-1.43 to -0.97) |
| **Subarachnoid hemorrhage** |  |  |  |  |  |  |  |  |  |  |
| **Location** | **1990** | | | | **2021** | | | | **EAPC (1990-2021)** | |
|  | **DALYs**  **NO. ×10^3^ (95% UI)** | **ASDR**  **NO. ×10^-5^ (95% UI)** | **Deaths**  **NO. ×10^3^ (95% UI)** | **ASMR**  **NO. ×10^-5^ (95% UI)** | **DALYs**  **NO. ×10^3^ (95% UI)** | **ASDR**  **NO. ×10^-5^ (95% UI)** | **Deaths**  **NO. ×10^3^ (95% UI)** | **ASMR**  **NO. ×10^-5^ (95% UI)** | **ASDR**  **NO. (95%CI)** | **ASMR**  **NO. (95%CI)** |
| Central Asia | 8.61 (3.94to16.69) | 17.2 (7.81to33.52) | 0.26 (0.12to0.52) | 0.58 (0.26to1.13) | 6.11 (2.52to13.36) | 6.87 (2.78to15.17) | 0.19 (0.07to0.43) | 0.25 (0.09to0.56) | -3.99 (-4.99 to -2.97) | -3.57 (-4.61 to -2.51) |
| Central Europe | 21.74 (6.71to54.48) | 15.02 (4.64to37.65) | 0.65 (0.2to1.64) | 0.45 (0.14to1.13) | 1.98 (0.06to13.09) | 1.06 (0.03to7.04) | 0.08 (0to0.51) | 0.04 (0to0.24) | -9.84 (-10.68 to -8.99) | -9.5 (-10.37 to -8.62) |
| Eastern Europe | 5.92 (1.1to24.19) | 2.22 (0.41to9.09) | 0.21 (0.04to0.85) | 0.08 (0.01to0.33) | 1.57 (0.19to7.48) | 0.5 (0.06to2.39) | 0.06 (0.01to0.29) | 0.02 (0to0.09) | -7.3 (-9.15 to -5.42) | -7.37 (-9.32 to -5.37) |
| Australasia | 0.02 (0to0.24) | 0.1 (0to1.06) | 0 (0to0.01) | 0 (0to0.04) | 0 (0to0.01) | 0 (0to0.02) | 0 (0to0) | 0 (0to0) | -11.22 (-11.89 to -10.54) | -11.15 (-11.8 to -10.49) |
| High-income Asia Pacific | 1.31 (0.1to6.73) | 0.64 (0.05to3.29) | 0.04 (0to0.21) | 0.02 (0to0.11) | 0.03 (0to0.18) | 0.01 (0to0.05) | 0 (0to0.01) | 0 (0to0) | -13.2 (-14.34 to -12.04) | -13.41 (-14.51 to -12.3) |
| High-income North America | 0.03 (0to0.24) | 0.01 (0to0.07) | 0 (0to0.01) | 0 (0to0) | 0.01 (0to0.03) | 0 (0to0.01) | 0 (0to0) | 0 (0to0) | -7.62 (-7.93 to -7.31) | -7.29 (-7.63 to -6.95) |
| Western Europe | 0.43 (0to3.41) | 0.09 (0to0.68) | 0.01 (0to0.12) | 0 (0to0.02) | 0.02 (0to0.19) | 0 (0to0.02) | 0 (0to0.01) | 0 (0to0) | -10.58 (-11.03 to -10.13) | -10.1 (-10.51 to -9.68) |
| Andean Latin America | 12.05 (5.85to18.93) | 49.07 (23.84to77.86) | 0.34 (0.16to0.53) | 1.52 (0.74to2.36) | 4.32 (0.83to13.1) | 6.88 (1.32to20.79) | 0.14 (0.03to0.4) | 0.22 (0.04to0.67) | -6.42 (-6.89 to -5.94) | -6.23 (-6.69 to -5.76) |
| Caribbean | 11.95 (7.29to16.87) | 42.26 (25.61to59.87) | 0.34 (0.2to0.49) | 1.25 (0.75to1.85) | 15.9 (8.36to24.96) | 30.57 (16.1to48.02) | 0.45 (0.23to0.72) | 0.84 (0.43to1.36) | -0.9 (-1.12 to -0.68) | -1.13 (-1.32 to -0.94) |
| Central Latin America | 15.71 (7.24to28.47) | 15.27 (6.96to27.82) | 0.42 (0.19to0.76) | 0.46 (0.21to0.84) | 13.85 (5.3to33.75) | 5.3 (2.03to12.97) | 0.43 (0.16to1.06) | 0.17 (0.06to0.42) | -3.48 (-3.72 to -3.24) | -3.2 (-3.5 to -2.91) |
| Southern Latin America | 5.98 (1.34to16.22) | 12.78 (2.87to34.68) | 0.18 (0.04to0.48) | 0.38 (0.08to1.04) | 0.32 (0to3.03) | 0.39 (0to3.69) | 0.01 (0to0.1) | 0.01 (0to0.12) | -11.28 (-11.59 to -10.98) | -10.98 (-11.28 to -10.69) |
| Tropical Latin America | 35.33 (17.62to61.33) | 31.02 (15.58to53.47) | 0.91 (0.46to1.56) | 0.88 (0.45to1.49) | 9.42 (2.05to26.44) | 3.57 (0.78to10.03) | 0.29 (0.06to0.8) | 0.11 (0.02to0.31) | -7.34 (-7.76 to -6.91) | -6.95 (-7.4 to -6.5) |
| North Africa and Middle East | 31.94 (13.28to58.63) | 16.92 (6.81to31.68) | 1.01 (0.39to1.94) | 0.63 (0.23to1.24) | 17.94 (8.87to31.15) | 3.3 (1.62to5.76) | 0.53 (0.25to0.96) | 0.11 (0.05to0.21) | -5.77 (-5.94 to -5.59) | -5.98 (-6.14 to -5.81) |
| Southeast Asia | 258.44 (181.96to375.83) | 88.27 (62.2to132.57) | 7.7 (5.27to12.01) | 3.14 (2.15to4.98) | 139.15 (59.04to263.59) | 19.48 (8.24to37.01) | 4.15 (1.74to8.07) | 0.65 (0.27to1.28) | -4.81 (-5.2 to -4.42) | -5.01 (-5.41 to -4.6) |
| South Asia | 544.79 (323.29to862.95) | 79.4 (46.39to129.54) | 15.79 (8.84to26.45) | 2.66 (1.44to4.64) | 555.94 (321.25to889.41) | 33.94 (19.62to54.39) | 16.87 (9.59to27.67) | 1.13 (0.64to1.85) | -2.82 (-3.02 to -2.62) | -2.87 (-3.06 to -2.68) |
| East Asia | 1888.14 (979.93to2600.73) | 218.4 (108.63to302.36) | 72.27 (33.35to100.86) | 10.1 (4.56to13.99) | 170.53 (46.42to486.62) | 8.02 (2.2to22.8) | 6.86 (1.78to19.87) | 0.33 (0.09to0.97) | -11.61 (-12.08 to -11.13) | -12.01 (-12.52 to -11.5) |
| Oceania | 5.04 (3.2to7.52) | 138.37 (89.11to206.13) | 0.14 (0.09to0.21) | 4.81 (3.08to7.37) | 8.42 (5.27to12.7) | 88.26 (55.41to131.51) | 0.22 (0.14to0.34) | 2.96 (1.78to4.5) | -1.47 (-1.51 to -1.42) | -1.59 (-1.62 to -1.55) |
| Central Sub-Saharan Africa | 11.93 (5.99to24.02) | 45.02 (22.23to92.93) | 0.33 (0.15to0.73) | 1.49 (0.63to3.4) | 23.72 (9.92to64.62) | 34.85 (14.67to94.88) | 0.64 (0.23to1.92) | 1.16 (0.41to3.45) | -0.97 (-1.1 to -0.83) | -0.94 (-1.09 to -0.79) |
| Eastern Sub-Saharan Africa | 54.22 (21.8to130.44) | 61.11 (24.31to149.9) | 1.52 (0.52to3.99) | 2.01 (0.67to5.44) | 84.43 (36.13to215.21) | 39.17 (16.88to99.35) | 2.22 (0.81to6.15) | 1.24 (0.46to3.5) | -1.65 (-1.71 to -1.59) | -1.76 (-1.83 to -1.7) |
| Southern Sub-Saharan Africa | 4.04 (2.36to6.4) | 13.12 (7.78to20.82) | 0.11 (0.07to0.19) | 0.41 (0.24to0.69) | 7.14 (4.3to11.28) | 10.53 (6.37to16.66) | 0.2 (0.12to0.32) | 0.33 (0.2to0.53) | **-0.42 (-0.9 to 0.07)** | **-0.4 (-0.93 to 0.13)** |
| Western Sub-Saharan Africa | 37.16 (17.51to82.15) | 36.33 (17.05to80.53) | 1.03 (0.45to2.43) | 1.15 (0.5to2.67) | 58.84 (29.27to125.67) | 23.2 (11.86to49.12) | 1.52 (0.7to3.47) | 0.71 (0.33to1.6) | -1.82 (-2.01 to -1.63) | -1.94 (-2.14 to -1.75) |
| HAP, household air pollution from solid fuels; DALYs, disability-adjusted life years; ASDR, age-standardized DALYs rate; ASMR, age-standardized deaths rate; EAPC, estimated annual percentage change; CI, confidential interval; UI, uncertainty interval | | | | | | | | | | |
