# Supplementary material for: Global burden, trends and health inequalities of stroke attributable to household air pollution, 1990–2021: a decomposition and prediction analysis
Source: Front Public Health. 2025 Sep 11;13:1625842. doi: 10.3389/fpubh.2025.1625842 (PMC12460407; doi:10.3389/fpubh.2025.1625842)
Supplement: Supplementary file 7 [file Table_3.docx]

| **Supplementary Table 3. Decomposition analysis for the global and regional burden of stroke and its subtypes attributable to HAP from 1990 to 2021.** | | | | | |
| --- | --- | --- | --- | --- | --- |
| **location** | **Cause** | **Overll difference** | **Aging** | **Population growth** | **Epidemiological change** |
| Global | Stroke | -10787897.44 | 4939556.33 (-45.79%) | 16246263.09 (-150.6%) | -31973716.86 (296.39%) |
| Global | Intracerebral hemorrhage | -7102314.88 | 2729957.13 (-38.44%) | 9897282.3 (-139.35%) | -19729554.31 (277.79%) |
| Global | Ischemic stroke | -1850406.54 | 1880003.28 (-101.6%) | 4860321.39 (-262.66%) | -8590731.21 (464.26%) |
| Global | Subarachnoid hemorrhage | -1835176.03 | 329595.91 (-17.96%) | 1488659.4 (-81.12%) | -3653431.34 (199.08%) |
| Low SDI | Stroke | 1570966.29 | -183332.18 (-11.67%) | 3635762.52 (231.43%) | -1881464.05 (-119.76%) |
| Low SDI | Intracerebral hemorrhage | 815824.1 | -136503.87 (-16.73%) | 2425385.71 (297.29%) | -1473057.74 (-180.56%) |
| Low SDI | Ischemic stroke | 657979.95 | -39940.21 (-6.07%) | 1013399.94 (154.02%) | -315479.78 (-47.95%) |
| Low SDI | Subarachnoid hemorrhage | 97162.25 | -6888.1 (-7.09%) | 196976.88 (202.73%) | -92926.54 (-95.64%) |
| Low-middle SDI | Stroke | 1093387.56 | 1011958.9 (92.55%) | 6171052.58 (564.4%) | -6089623.92 (-556.95%) |
| Low-middle SDI | Intracerebral hemorrhage | 333190.54 | 533315.15 (160.06%) | 3829073.19 (1149.21%) | -4029197.8 (-1209.28%) |
| Low-middle SDI | Ischemic stroke | 756099.76 | 435630.95 (57.62%) | 1863294.87 (246.44%) | -1542826.06 (-204.05%) |
| Low-middle SDI | Subarachnoid hemorrhage | 4097.26 | 43012.79 (1049.79%) | 478684.52 (11683.04%) | -517600.06 (-12632.84%) |
| Middle SDI | Stroke | -7847830.31 | 3535219.11 (-45.05%) | 7605692.92 (-96.91%) | -18988742.34 (241.96%) |
| Middle SDI | Intracerebral hemorrhage | -4925017.79 | 1969621.04 (-39.99%) | 4542374.36 (-92.23%) | -11437013.2 (232.22%) |
| Middle SDI | Ischemic stroke | -1539295.9 | 1220368.22 (-79.28%) | 2157546.6 (-140.16%) | -4917210.73 (319.45%) |
| Middle SDI | Subarachnoid hemorrhage | -1383516.61 | 345229.84 (-24.95%) | 905771.96 (-65.47%) | -2634518.42 (190.42%) |
| High-middle SDI | Stroke | -5220579.19 | 1071069.79 (-20.52%) | 1877991.16 (-35.97%) | -8169640.13 (156.49%) |
| High-middle SDI | Intracerebral hemorrhage | -3157830.09 | 575625.35 (-18.23%) | 1085256.28 (-34.37%) | -4818711.73 (152.6%) |
| High-middle SDI | Ischemic stroke | -1538370.81 | 421538.83 (-27.4%) | 625570.26 (-40.66%) | -2585479.89 (168.07%) |
| High-middle SDI | Subarachnoid hemorrhage | -524378.29 | 73905.6 (-14.09%) | 167164.62 (-31.88%) | -765448.51 (145.97%) |
| High SDI | Stroke | -378110.5 | 64165.15 (-16.97%) | 90365.93 (-23.9%) | -532641.58 (140.87%) |
| High SDI | Intracerebral hemorrhage | -166079.79 | 22937.86 (-13.81%) | 38649.15 (-23.27%) | -227666.8 (137.08%) |
| High SDI | Ischemic stroke | -183766.22 | 38592.64 (-21%) | 45271.12 (-24.64%) | -267629.99 (145.64%) |
| High SDI | Subarachnoid hemorrhage | -28264.49 | 2634.65 (-9.32%) | 6445.66 (-22.8%) | -37344.8 (132.13%) |
| HAP, household air pollution from solid fuels; SDI, socio-demographic index; | | | | | |
