# Supplementary material for: Global burden, trends and health inequalities of stroke attributable to household air pollution, 1990–2021: a decomposition and prediction analysis
Source: Front Public Health. 2025 Sep 11;13:1625842. doi: 10.3389/fpubh.2025.1625842 (PMC12460407; doi:10.3389/fpubh.2025.1625842)
Supplement: Supplementary file 8 [file Table_4.docx]

| **Supplementary Table 4. The slope index for cross-national inequality analysis of stroke and its subtypes attributable to HAP.** | | | | | | | | |
| --- | --- | --- | --- | --- | --- | --- | --- | --- |
| **Cause** | **Year** | **lm** | **lm_lower** | **lm_upper** | **ncvTest_p** | **rlm** | **rlm_lower** | **rlm_upper** |
| Stroke | 1990 | -820.968211 | -944.461197 | -697.475225 | 6.43e-05 | -769.939267 | -856.860822 | -683.017713 |
| Stroke | 2021 | -646.922136 | -738.259544 | -555.584728 | 7.53e-17 | -551.595460 | -613.139329 | -490.051590 |
| Intracerebral hemorrhage | 1990 | -582.860035 | -668.112907 | -497.607163 | 4.84e-08 | -522.931420 | -578.561927 | -467.300913 |
| Intracerebral hemorrhage | 2021 | -421.959296 | -484.166783 | -359.751808 | 5.01e-19 | -344.617271 | -383.745634 | -305.488908 |
| Ischemic stroke | 1990 | -197.660681 | -237.923691 | -157.397671 | 7.01e-01 | -202.266647 | -230.861539 | -173.671755 |
| Ischemic stroke | 2021 | -189.192201 | -216.818219 | -161.566184 | 1.50e-11 | -170.868177 | -190.345980 | -151.390374 |
| Subarachnoid hemorrhage | 1990 | -40.447495 | -51.013574 | -29.881416 | 2.11e-05 | -31.901392 | -36.797641 | -27.005143 |
| Subarachnoid hemorrhage | 2021 | -35.770639 | -42.341028 | -29.200250 | 3.87e-24 | -24.629385 | -27.440524 | -21.818246 |
| HAP, household air pollution from solid fuels | | | | | | | | |
