# Supplementary material for: Global burden, trends and health inequalities of stroke attributable to household air pollution, 1990–2021: a decomposition and prediction analysis
Source: Front Public Health. 2025 Sep 11;13:1625842. doi: 10.3389/fpubh.2025.1625842 (PMC12460407; doi:10.3389/fpubh.2025.1625842)
Supplement: Supplementary file 9 [file Table_5.docx]

| **Supplementary Table 5. The concentration index for cross-national inequality analysis of stroke and its subtypes attributable to HAP.** | | | | | | |
| --- | --- | --- | --- | --- | --- | --- |
| **Cause** | **Year** | **Concentration_index** | **UCI** | **LCI** | **SE** | **P** |
| Stroke | 1990 | -0.230015 | -0.170178 | -0.289852 | 0.030529 | <0.001 |
| Stroke | 2021 | -0.376693 | -0.311991 | -0.441395 | 0.033011 | <0.001 |
| Intracerebral hemorrhage | 1990 | -0.253842 | -0.193202 | -0.314482 | 0.030939 | <0.001 |
| Intracerebral hemorrhage | 2021 | -0.396928 | -0.327778 | -0.466079 | 0.035281 | <0.001 |
| Ischemic stroke | 1990 | -0.206136 | -0.150032 | -0.262240 | 0.028624 | <0.001 |
| Ischemic stroke | 2021 | -0.338819 | -0.276661 | -0.400977 | 0.031713 | <0.001 |
| Subarachnoid hemorrhage | 1990 | -0.151778 | -0.069503 | -0.234054 | 0.041977 | <0.001 |
| Subarachnoid hemorrhage | 2021 | -0.395137 | -0.324457 | -0.465816 | 0.036061 | <0.001 |
| HAP, household air pollution from solid fuels | | | | | | |
