# Supplementary material for: Global burden, trends and health inequalities of stroke attributable to household air pollution, 1990–2021: a decomposition and prediction analysis
Source: Front Public Health. 2025 Sep 11;13:1625842. doi: 10.3389/fpubh.2025.1625842 (PMC12460407; doi:10.3389/fpubh.2025.1625842)
Supplement: Supplementary file 10 [file Table_6.docx]

| **Supplementary Table 6. Projections of ASDR for stroke and its subtypes attributable to HAP.** | | | | | |
| --- | --- | --- | --- | --- | --- |
| **year** | **sex** | **measure** | **location** | **cause** | **Per 100000 population (95% UI)** |
| 2022 | Both | DALYs (Disability-Adjusted Life Years) | Global | Stroke | 205.08 (190.05to220.1) |
| 2023 | Both | DALYs (Disability-Adjusted Life Years) | Global | Stroke | 204.35 (185.45to223.25) |
| 2024 | Both | DALYs (Disability-Adjusted Life Years) | Global | Stroke | 203.87 (179.1to228.63) |
| 2025 | Both | DALYs (Disability-Adjusted Life Years) | Global | Stroke | 203.73 (171.51to235.96) |
| 2026 | Both | DALYs (Disability-Adjusted Life Years) | Global | Stroke | 203.82 (162.82to244.82) |
| 2027 | Both | DALYs (Disability-Adjusted Life Years) | Global | Stroke | 204 (153.06to254.94) |
| 2028 | Both | DALYs (Disability-Adjusted Life Years) | Global | Stroke | 204.41 (142.43to266.39) |
| 2029 | Both | DALYs (Disability-Adjusted Life Years) | Global | Stroke | 205.21 (131.04to279.38) |
| 2030 | Both | DALYs (Disability-Adjusted Life Years) | Global | Stroke | 206.51 (118.89to294.12) |
| 2031 | Both | DALYs (Disability-Adjusted Life Years) | Global | Stroke | 208.19 (105.78to310.6) |
| 2032 | Both | DALYs (Disability-Adjusted Life Years) | Global | Stroke | 210.17 (91.5to328.84) |
| 2033 | Both | DALYs (Disability-Adjusted Life Years) | Global | Stroke | 212.6 (75.97to349.23) |
| 2034 | Both | DALYs (Disability-Adjusted Life Years) | Global | Stroke | 215.63 (59to372.27) |
| 2035 | Both | DALYs (Disability-Adjusted Life Years) | Global | Stroke | 219.38 (40.41to398.47) |
| 2022 | Both | DALYs (Disability-Adjusted Life Years) | Global | Intracerebral hemorrhage | 118.92 (107.64to130.19) |
| 2023 | Both | DALYs (Disability-Adjusted Life Years) | Global | Intracerebral hemorrhage | 118.04 (104.58to131.49) |
| 2024 | Both | DALYs (Disability-Adjusted Life Years) | Global | Intracerebral hemorrhage | 117.31 (100.46to134.15) |
| 2025 | Both | DALYs (Disability-Adjusted Life Years) | Global | Intracerebral hemorrhage | 116.77 (95.53to138.01) |
| 2026 | Both | DALYs (Disability-Adjusted Life Years) | Global | Intracerebral hemorrhage | 116.38 (89.9to142.85) |
| 2027 | Both | DALYs (Disability-Adjusted Life Years) | Global | Intracerebral hemorrhage | 116.1 (83.65to148.54) |
| 2028 | Both | DALYs (Disability-Adjusted Life Years) | Global | Intracerebral hemorrhage | 116 (76.9to155.1) |
| 2029 | Both | DALYs (Disability-Adjusted Life Years) | Global | Intracerebral hemorrhage | 116.14 (69.67to162.61) |
| 2030 | Both | DALYs (Disability-Adjusted Life Years) | Global | Intracerebral hemorrhage | 116.56 (61.95to171.17) |
| 2031 | Both | DALYs (Disability-Adjusted Life Years) | Global | Intracerebral hemorrhage | 117.21 (53.62to180.79) |
| 2032 | Both | DALYs (Disability-Adjusted Life Years) | Global | Intracerebral hemorrhage | 118.08 (44.59to191.56) |
| 2033 | Both | DALYs (Disability-Adjusted Life Years) | Global | Intracerebral hemorrhage | 119.24 (34.75to203.73) |
| 2034 | Both | DALYs (Disability-Adjusted Life Years) | Global | Intracerebral hemorrhage | 120.77 (23.99to217.58) |
| 2035 | Both | DALYs (Disability-Adjusted Life Years) | Global | Intracerebral hemorrhage | 122.72 (12.28to233.44) |
| 2022 | Both | DALYs (Disability-Adjusted Life Years) | Global | Ischemic stroke | 72.53 (67.86to77.2) |
| 2023 | Both | DALYs (Disability-Adjusted Life Years) | Global | Ischemic stroke | 72.73 (66.5to78.96) |
| 2024 | Both | DALYs (Disability-Adjusted Life Years) | Global | Ischemic stroke | 73.01 (64.47to81.55) |
| 2025 | Both | DALYs (Disability-Adjusted Life Years) | Global | Ischemic stroke | 73.42 (61.97to84.87) |
| 2026 | Both | DALYs (Disability-Adjusted Life Years) | Global | Ischemic stroke | 73.92 (59.05to88.79) |
| 2027 | Both | DALYs (Disability-Adjusted Life Years) | Global | Ischemic stroke | 74.42 (55.68to93.17) |
| 2028 | Both | DALYs (Disability-Adjusted Life Years) | Global | Ischemic stroke | 74.99 (51.91to98.06) |
| 2029 | Both | DALYs (Disability-Adjusted Life Years) | Global | Ischemic stroke | 75.69 (47.82to103.56) |
| 2030 | Both | DALYs (Disability-Adjusted Life Years) | Global | Ischemic stroke | 76.6 (43.39to109.82) |
| 2031 | Both | DALYs (Disability-Adjusted Life Years) | Global | Ischemic stroke | 77.69 (38.55to116.84) |
| 2032 | Both | DALYs (Disability-Adjusted Life Years) | Global | Ischemic stroke | 78.9 (33.19to124.61) |
| 2033 | Both | DALYs (Disability-Adjusted Life Years) | Global | Ischemic stroke | 80.29 (27.26to133.32) |
| 2034 | Both | DALYs (Disability-Adjusted Life Years) | Global | Ischemic stroke | 81.94 (20.69to143.19) |
| 2035 | Both | DALYs (Disability-Adjusted Life Years) | Global | Ischemic stroke | 83.91 (13.35to154.49) |
| 2022 | Both | DALYs (Disability-Adjusted Life Years) | Global | Subarachnoid hemorrhage | 13.05 (11.9to14.19) |
| 2023 | Both | DALYs (Disability-Adjusted Life Years) | Global | Subarachnoid hemorrhage | 13.09 (11.63to14.54) |
| 2024 | Both | DALYs (Disability-Adjusted Life Years) | Global | Subarachnoid hemorrhage | 13.14 (11.22to15.07) |
| 2025 | Both | DALYs (Disability-Adjusted Life Years) | Global | Subarachnoid hemorrhage | 13.22 (10.7to15.75) |
| 2026 | Both | DALYs (Disability-Adjusted Life Years) | Global | Subarachnoid hemorrhage | 13.33 (10.09to16.58) |
| 2027 | Both | DALYs (Disability-Adjusted Life Years) | Global | Subarachnoid hemorrhage | 13.46 (9.38to17.53) |
| 2028 | Both | DALYs (Disability-Adjusted Life Years) | Global | Subarachnoid hemorrhage | 13.61 (8.6to18.63) |
| 2029 | Both | DALYs (Disability-Adjusted Life Years) | Global | Subarachnoid hemorrhage | 13.8 (7.73to19.87) |
| 2030 | Both | DALYs (Disability-Adjusted Life Years) | Global | Subarachnoid hemorrhage | 14.03 (6.77to21.29) |
| 2031 | Both | DALYs (Disability-Adjusted Life Years) | Global | Subarachnoid hemorrhage | 14.3 (5.7to22.89) |
| 2032 | Both | DALYs (Disability-Adjusted Life Years) | Global | Subarachnoid hemorrhage | 14.61 (4.49to24.72) |
| 2033 | Both | DALYs (Disability-Adjusted Life Years) | Global | Subarachnoid hemorrhage | 14.96 (3.13to26.8) |
| 2034 | Both | DALYs (Disability-Adjusted Life Years) | Global | Subarachnoid hemorrhage | 15.38 (1.61to29.17) |
| 2035 | Both | DALYs (Disability-Adjusted Life Years) | Global | Subarachnoid hemorrhage | 15.86 (0.22to31.92) |
| HAP, household air pollution from solid fuels | | | | | |
